# Post-therapy imaging of adult brain tumours: patterns, pitfalls, and practical framework

**DOI:** 10.1186/s13244-026-02398-y

**Published:** 2026-09-19

**Authors:** Mina F. G. Isaac

**Affiliations:** Department of Radiology, Belfast Health and Social Care Trust, Belfast, UK

**Keywords:** Brain neoplasms, Chemoradiotherapy, Immunotherapy, Radiation injuries, Radiosurgery

## Abstract

**Abstract:**

Post-therapy imaging of adult brain tumours is among the most challenging tasks in neuroradiology. Distinguishing true tumour progression from treatment-related changes—including pseudoprogression, radiation necrosis, and atypical immunotherapy responses—requires integration of imaging timing, morphology, and clinical context. This review synthesises current literature into a practical, treatment-based framework, addressing gliomas and metastases separately and, within each, organising findings by treatment modality—surgical resection, chemoradiotherapy, stereotactic radiosurgery, systemic therapy (targeted and anti-angiogenic agents, and immunotherapy), and locoregional techniques. For each treatment context, we outline expected imaging evolution, characteristic patterns of treatment effect, key features differentiating these from tumour progression, and the role of MRI and nuclear medicine techniques. The review concludes with a practical reporting framework built around treatment received and time since treatment, integrating clinical context, imaging pattern, and interval change to support radiology reporting across diverse therapeutic combinations.

**Critical relevance statement:**

Recognising treatment-specific imaging pitfalls—pseudoprogression, pseudoresponse, radiation injury, and immune-related changes—is essential for accurate response assessment and prevents misdirected management in neuro-oncology.

**Key Points:**

- Distinguishing true tumour progression from treatment-related change on post-therapy brain MRI is a frequent diagnostic challenge, because findings vary with treatment type and timing.
- Pseudoprogression is context-dependent—its definition, timing, and morphology differ across chemoradiotherapy, IDH-mutant glioma, stereotactic radiosurgery, and immunotherapy, so no single rule fits all settings.

**Graphical Abstract:**

**Figure:**
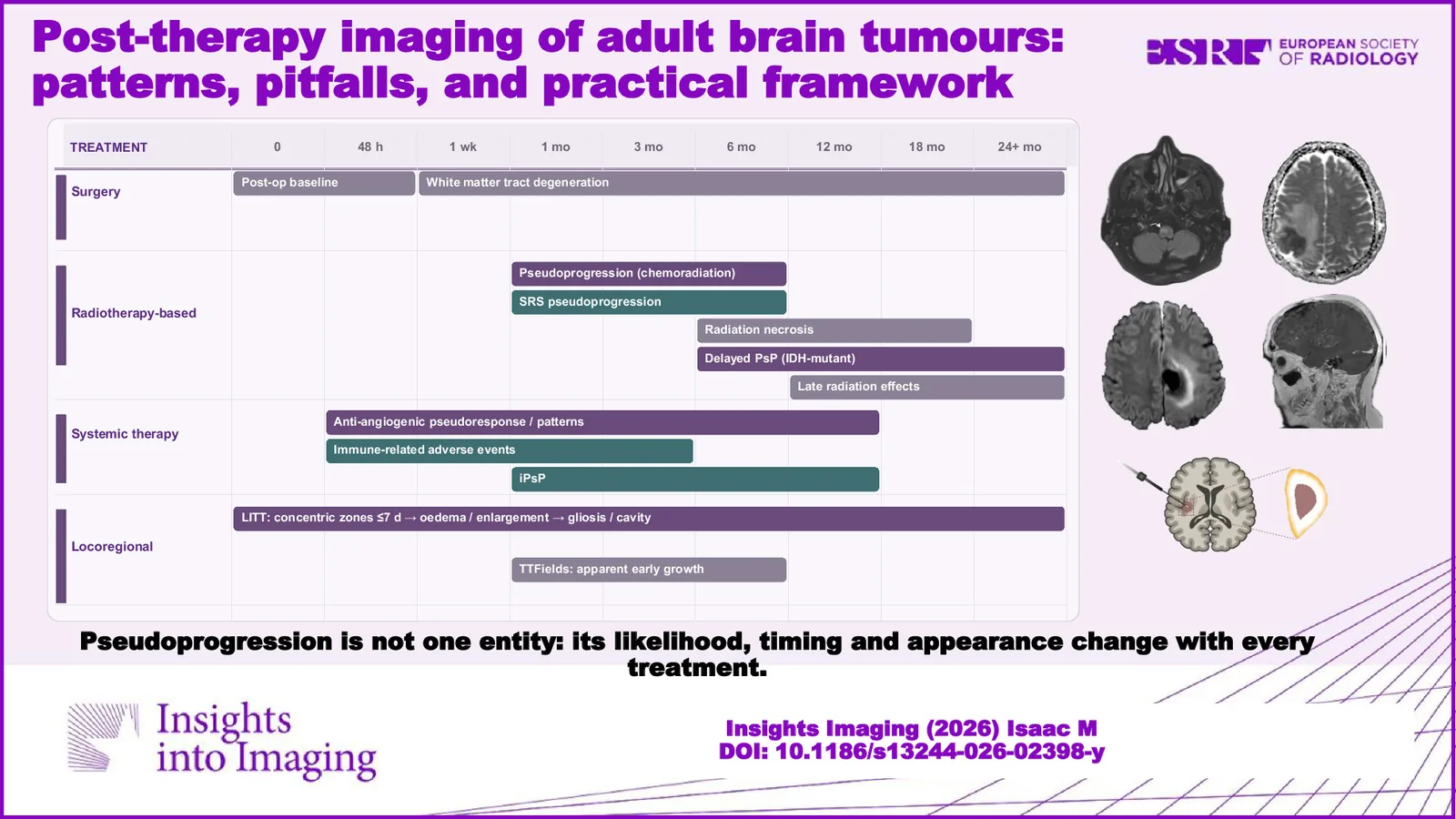

## Introduction

Modern management of adult brain tumours combines maximal safe resection, concurrent chemoradiotherapy, stereotactic radiosurgery (SRS), immunotherapy, anti-angiogenic and other targeted systemic agents, and an expanding range of locoregional techniques. These advances have widened the spectrum of treatment-related imaging changes that can mimic progression. Accurate post-therapy interpretation therefore depends less on isolated MRI signs than on the integration of treatment type, time from therapy, and pattern of evolution.

Response assessment frameworks have evolved alongside these challenges, from the Macdonald criteria to RANO and its context-specific adaptations. RANO 2.0 provides a unified framework for adult gliomas, iRANO addresses response assessment during immunotherapy, and RANO-BM applies to brain metastases.

This review focuses on adult brain tumours; paediatric neoplasms are outside its scope because they differ in biology, treatment, and imaging. Adult diffuse gliomas and brain metastases are presented separately and organised by treatment modality and timing. Shared diagnostic tools and treatment-related changes applicable across both disease groups are described once and cross-referenced in the other section.

## Section I: Treatment-related imaging changes in adult diffuse gliomas

### Postoperative imaging changes

This section focuses on imaging changes specific to tumour resection. General postoperative complications such as haemorrhage and infection are not addressed.

The resection cavity typically contains cerebrospinal fluid (CSF) and blood products in variable proportions; its appearance evolves over time, complicating both follow-up interpretation and radiotherapy target volume definition [1]. Current ESTRO–EANO guidelines therefore recommend a dedicated post-surgical MRI within 2 weeks before starting radiotherapy to improve planning accuracy [2].

#### Residual tumour and enhancement

Residual enhancing or non-enhancing tumour should be assessed against the preoperative study. In glioblastoma, the extent of resection may be categorised using the RANO resect framework, ranging from supramaximal contrast-enhancing resection to biopsy only [3]. Peritumoural oedema may persist early after surgery and obscure tumour boundaries, requiring correlation with contrast-enhanced and preoperative imaging. Under RANO 2.0, in the newly diagnosed setting, the post-radiotherapy MRI—rather than the immediate postoperative study—serves as the baseline for response assessment [4].

Enhancement adjacent to the cavity may reflect reactive changes or residual tumour [5]. Reactive enhancement increases substantially after 45 h, supporting the recommendation for early postoperative MRI within a 48-h window [5, 6]. Thin, smooth linear enhancement and dural enhancement beneath the craniotomy are usually reactive, whereas nodular, thick, or asymmetric enhancement corresponding to the preoperative tumour favours residual disease.

#### Diffusion abnormalities

Postoperative infarcts are detected on DWI in up to 44% of glioma resections [7]. Thin rim-shaped restriction along the cavity margin, particularly when less than 3 mm thick, usually reflects marginal microischaemic injury [7–9]. Sector-shaped infarcts extend as wedge-shaped areas into adjacent parenchyma, particularly after temporal lobe resection, and may involve deep white matter through medullary artery injury [7, 10].

**Fig. 1:**
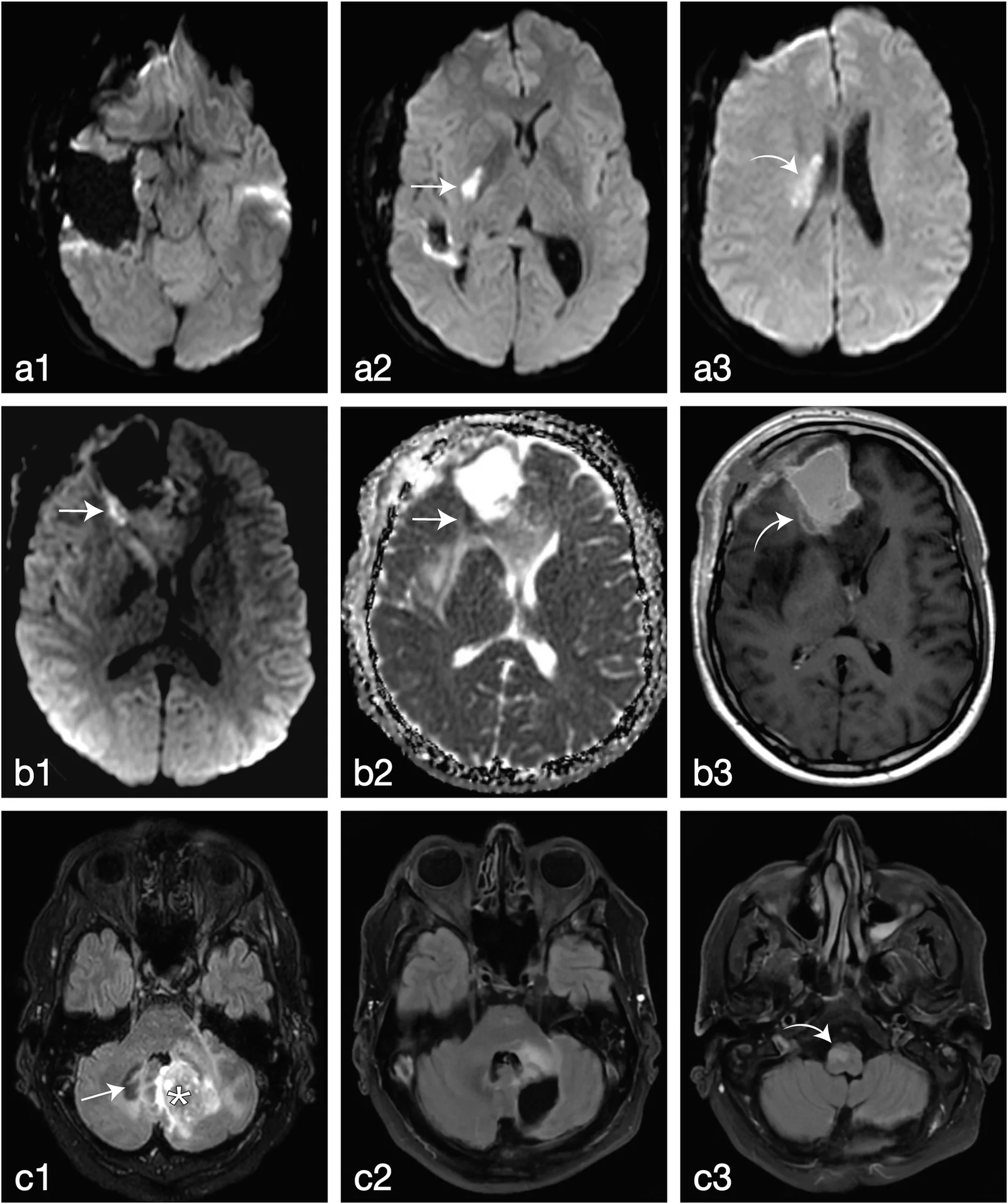
Post-surgical changes and pitfalls after brain tumour resection. Three patients illustrating post-surgical changes that may mimic or obscure residual tumour. Case 1—remote infarction (**a1**–**a3**). After right temporal glioma resection. **a1** Axial DWI showing the resection cavity. **a2**, **a3** More cranial DWI showing restricted diffusion in the posterior limb of the internal capsule (straight arrow) and periventricular white matter (curved arrow), in the anterior choroidal artery territory. Case 2—subacute infarct mimicking tumour (**b1**–**b3**). After frontal resection. **b1** DWI showing restricted diffusion at the posterolateral cavity margin (straight arrow). **b2** ADC map confirming low signal (straight arrow). **b3** Post-contrast T1 showing peripheral enhancement (curved arrow)—a pitfall mimicking residual tumour. Case 3—hypertrophic olivary degeneration (**c1**–**c3**). After posterior fossa resection. **c1** Preoperative FLAIR: left cerebellar tumour involving the left dentate nucleus (asterisk); right dentate for comparison (straight arrow). **c2** Postoperative FLAIR: resection cavity. **c3** Follow-up FLAIR showing right inferior olivary hyperintensity (curved arrow)

Rim-type infarcts predominate after glioma resection, likely reflecting retraction-related marginal injury; in comparative studies, sector-type infarcts were more frequent after meningioma surgery, probably because of cortical vessel injury during tumour detachment [8]. Remote infarcts distant from the surgical bed may also occur (Fig. 1a1–a3). A key pitfall is subacute infarction near the resection margin, which may enhance and mimic tumour (Fig. 1b1–b3); correlation with diffusion-weighted imaging (DWI) is essential.

#### BCNU wafer implantation

Carmustine wafers appear on early postoperative MRI as thin linear signal loss foci, usually without enhancement [11]. Some patients develop early cystic cavity enlargement, which may show rim enhancement and diffusion restriction, mimicking abscess or recurrence [12]. A transient increase in the surrounding FLAIR signal within the first several weeks likely reflects benign inflammatory change [13].

#### Wallerian and trans-synaptic degeneration

Surgery may also produce delayed Wallerian or trans-synaptic degeneration. Wallerian degeneration evolves from early diffusion abnormality to T2/FLAIR hyperintensity and later tract atrophy. Following posterior fossa surgery, disruption of the dentato-rubro-olivary pathway may produce hypertrophic olivary degeneration, a rare but characteristic trans-synaptic phenomenon that can be mistaken for tumour (Fig. 1c1–c3) [14].

### Imaging changes after radiotherapy-based treatment

#### Pseudoprogression after Stupp-protocol chemoradiotherapy for IDH-wildtype glioblastoma

Following resection, patients with newly diagnosed isocitrate dehydrogenase (IDH)–wildtype glioblastoma and adequate performance status typically receive the Stupp protocol: fractionated radiotherapy with concurrent daily temozolomide followed by adjuvant temozolomide [15]. This regimen is also used for IDH-mutant astrocytoma, WHO grade 4 [16]. O⁶-methylguanine-DNA methyltransferase (MGMT) promoter methylation increases temozolomide sensitivity and is associated with a higher likelihood of pseudoprogression [17–19].

**Fig. 2:**
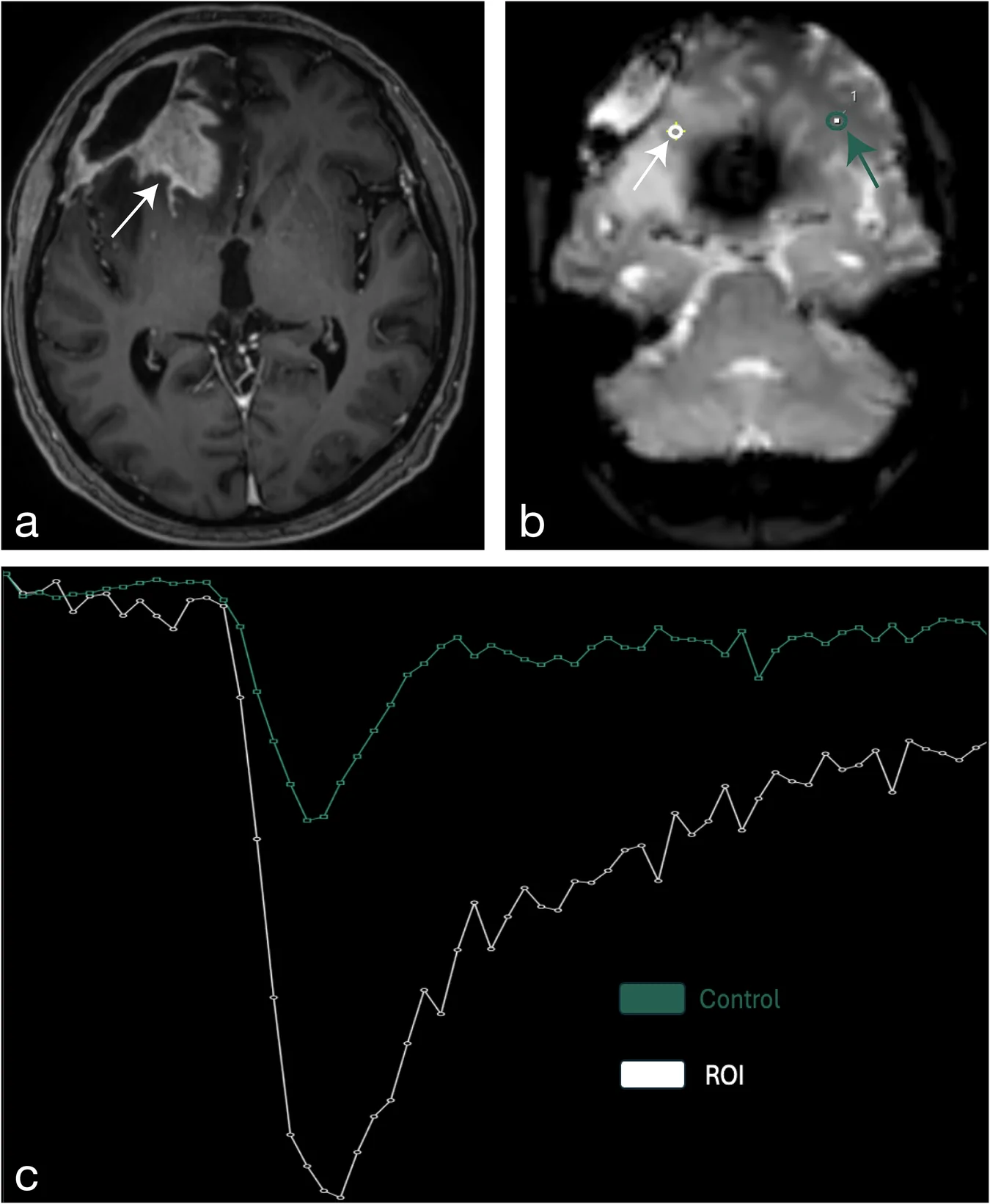
Dynamic susceptibility contrast perfusion in the differentiation of true progression from pseudoprogression. A patient with right frontal glioblastoma scanned in the early post-chemoradiotherapy period, presenting with new contrast-enhancing tissue raising the differential of pseudoprogression versus true tumour progression. **a** Axial post-contrast T1-weighted image showing a thick irregular enhancing component adjacent to the resection cavity (white arrow). **b** Dynamic susceptibility contrast (DSC) source image showing the two regions of interest used for perfusion analysis: a lesion ROI placed on the enhancing component (white arrow) and a control ROI placed in contralateral normal-appearing white matter (teal green arrow). **c** Corresponding time–signal intensity curves: the lesion ROI (white curve) shows a marked signal drop with delayed and reduced post-bolus signal recovery, in contrast to the smaller drop and prompt return to baseline of the control ROI (teal green curve)—a pattern indicating elevated relative cerebral blood volume (rCBV) with low percentage of signal recovery (PSR) in the lesion, favouring true tumour progression over pseudoprogression

Pseudoprogression (PsP) here refers to new or increasing contrast enhancement within the irradiated field that subsequently stabilises or regresses without a change in therapy. It most commonly occurs within the first 12 weeks after chemoradiotherapy but may arise during the first 3–6 months [20, 21]. It reflects treatment-induced endothelial injury, blood–brain barrier (BBB) disruption, and local inflammation rather than neoplastic growth. Radiation necrosis may have overlapping imaging appearances but generally develops later, most often beyond 6 months, and is more likely to persist [22, 23]. Conventional MRI patterns described in PsP and radiation necrosis include the soap-bubble or Swiss-cheese appearance and the wavefront sign of ill-defined undulating margins, but none reliably distinguishes treatment effect from tumour progression [24]. Beyond conventional MRI, several techniques have been studied; their relative roles, signal directions, and key considerations are summarised in Table 1. Dynamic susceptibility contrast perfusion is the most widely applied of these in routine practice (Fig. 2).

**Table 1:** Advanced imaging techniques for distinguishing tumour progression from treatment effect [81–101]

Technique	Measures	Favours tumour progression	Favours treatment effect	Key considerations
Diffusion				
DWI/ADC	Water mobility as a surrogate of cellularity	Lower ADC	Higher ADC	Mean ADC may be falsely elevated by necrosis or cystic change; lower-percentile, for example, 5th-percentile, ADC may improve sensitivity [81, 82].
IVIM	True diffusion Pseudodiffusion Perfusion fraction Standard ADC assumes Gaussian diffusion and does not distinguish true molecular diffusion from microvascular perfusion-related pseudodiffusion. IVIM separates these components.	Decreased true diffusion. Increased pseudodiffusion. Increased perfusion fraction.	Opposite pattern	Limited availability and validation [83].
DKI	Non-Gaussian water motion and tissue complexity	Higher mean kurtosis	Lower mean kurtosis	Specialised acquisition and postprocessing [84].
Perfusion				
DSC	rCBV from first-pass T2* signal loss	Elevated rCBV	Lower rCBV	Needs leakage correction^a^· prone to susceptibility artefacts^b^· automated calibration may improve reproducibility. rCBV may be paradoxically low in recurrence within irradiated tissue [85–88].
VAI	Microvessel architecture from dual-echo DSC Compares GRE signal, sensitive to all vessel calibres, with SE signal, weighted toward capillaries.	Early progression shows venous dominance, with SE preceding GRE and a counterclockwise hysteresis loop.	Pseudoprogression shows arterial dominance, with GRE preceding SE and a clockwise loop.	Research-grade; limited clinical application [89].
DCE	Vascular permeability and plasma volume	Elevated Ktrans, often with increased plasma volume and initial area under the enhancement curve	Lower Ktrans	Less susceptibility artefact than DSC; longer acquisition and complex postprocessing; less standardised Ktrans thresholds [85, 90, 91].
ASL	Cerebral blood flow without contrast	Higher CBF	Lower CBF	Useful when gadolinium contraindicated; lower signal-to-noise ratio [86, 92, 93].
Spectroscopy and molecular MRI				
MRS	Tissue metabolite concentrations	Increased Cho/NAA or Cho/Cr.	Lipid peak; no substantial Cho rise.	Cho elevation is nonspecific and overlap limits specificity; emerging ratios, including lower mI/Cr and higher Lac/Glx, may improve discrimination [94, 95].
APT-CEST	Mobile protein content via amide–water proton exchange	Increased APT signal	Hypo- to isointense APT signal	Also raised in meningiomas and lymphomas. Pooled sensitivity is approximately 88% and specificity approximately 84% in gliomas [96–99].
Nuclear medicine				
Amino-acid PET (^18^F-FET)	Amino-acid tracer uptake	High tumour-to-background uptake	Low uptake	Greater lesion-to-background contrast than FDG-PET; emerging hypoxia, neuroinflammation, and theranostic tracers have evolving clinical roles [100].
SPECT (^201^Tl or ^99m^Tc-MIBI)	Radiotracer uptake	Increased uptake	Low uptake	High specificity; limited spatial resolution [85].
Computational imaging				
Radiomics/AI	Machine-learning integration of multiparametric MRI and clinical data	Model-dependent	Model-dependent	Promising for pseudoprogression versus progression; multicentre validation remains limited, and AI-RANO does not yet support routine or trial adoption [101].

The general principles of advanced imaging apply across post-treatment settings, but most discriminators in this table derive from post-chemoradiation high-grade glioma. Extrapolation to other settings should be cautious, as pathophysiology and thresholds differ; for example, relative cerebral blood volume may be higher in immunotherapy-related pseudoprogression than in post-chemoradiation pseudoprogression

*ADC* apparent diffusion coefficient, *AI* artificial intelligence, *APT* amide proton transfer, *ASL* arterial spin labelling, *CBF* cerebral blood flow, *CEST* chemical exchange saturation transfer, *Cho* choline, *Cr* creatine, *DCE* dynamic contrast-enhanced, *DKI* diffusion kurtosis imaging, *DSC* dynamic susceptibility contrast, *DWI* diffusion-weighted imaging, *FDG* fluorodeoxyglucose, *FET* fluoroethyltyrosine, *Glx* glutamate–glutamine complex, *GRE* gradient echo, *IVIM* intravoxel incoherent motion, *Ktrans* volume transfer constant, *Lac* lactate, *mI* myo-inositol, *MIBI* methoxyisobutylisonitrile, *MRS* magnetic resonance spectroscopy, *NAA* N-acetylaspartate, *PET* positron emission tomography, *rCBV* relative cerebral blood volume, *SE* spin echo, *SPECT* single-photon emission computed tomography, *TP* true progression, *VAI* vessel architectural imaging

^a^ Leakage correction includes preload dosing, optimised acquisition, and postprocessing to reduce blood–brain barrier leakage-related rCBV error

^b^ Susceptibility artefacts from blood products, calcification, or postoperative air may degrade DSC interpretation

#### Delayed pseudoprogression after radiotherapy-based treatment in IDH-mutant gliomas

Pseudoprogression in IDH-mutant gliomas is increasingly recognised as a distinct entity. Although it shares the same broad mechanisms as IDH-wildtype pseudoprogression—treatment-related vascular injury, blood–brain barrier disruption, and inflammation—its timing and morphology are different (Table 3).

Reported incidence is approximately 16–19% [25, 26], with a strong association with radiotherapy exposure [25]. In the POLA network cohort, pseudoprogression occurred more often after radiotherapy plus procarbazine, lomustine, and vincristine (PCV) (26%) than after radiotherapy plus temozolomide (8%) or radiotherapy alone (7%) [26].

**Fig. 3:**
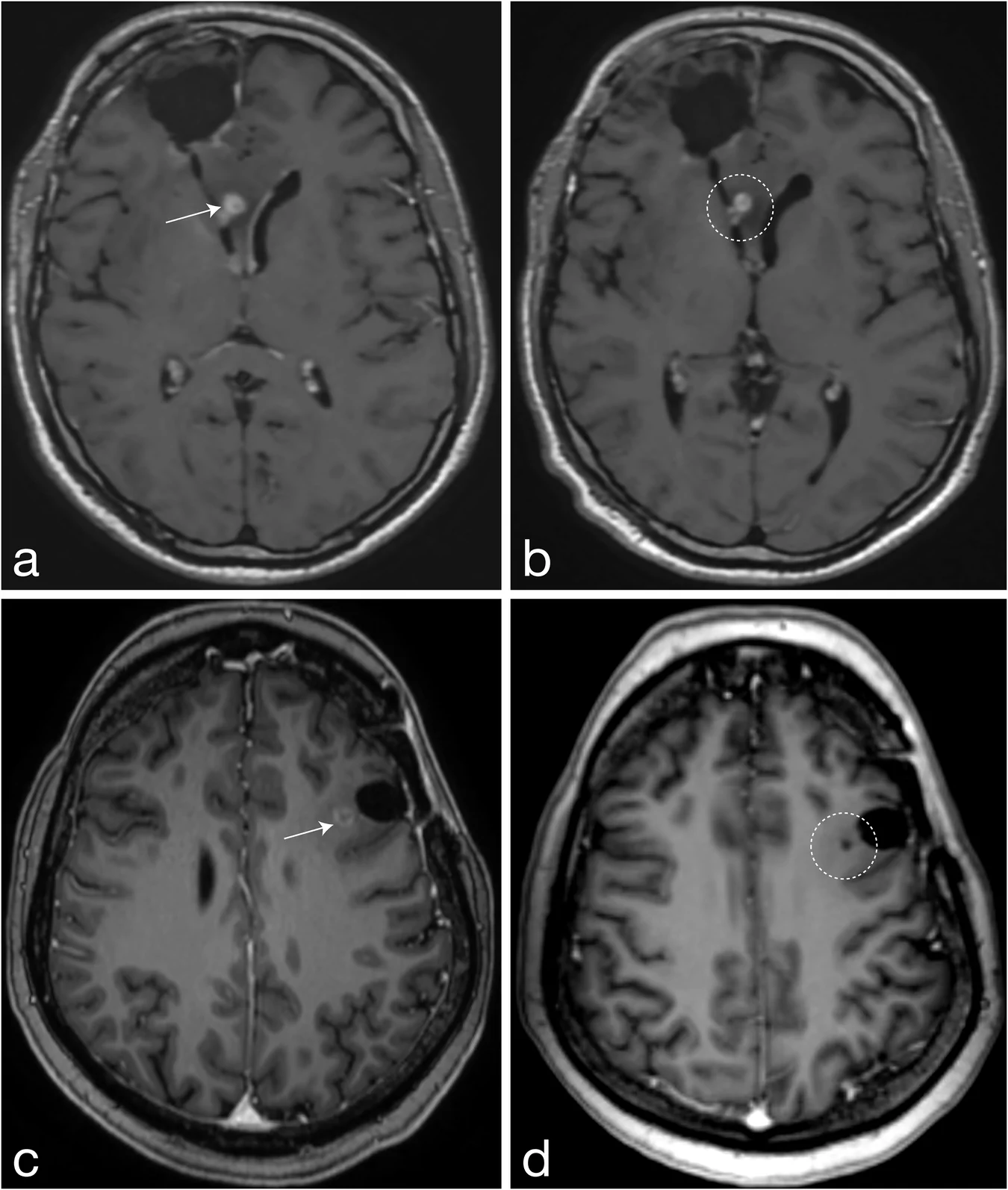
Delayed pseudoprogression in IDH-mutant glioma. Top row (Case 1): a patient with IDH-mutant astrocytoma treated with surgical debulking, radiotherapy and adjuvant PCV chemotherapy. Axial post-contrast T1-weighted images at (**a**) 6 months and (**b**) 9 months after completion of radiotherapy demonstrate a small punctate periventricular enhancing focus (arrow in(**a**; dashed circle in **b**) that remained essentially unchanged, possibly marginally smaller, on follow-up. Bottom row (Case 2): a patient with IDH-mutant glioma treated with biopsy, radiotherapy and adjuvant PCV chemotherapy. Axial post-contrast T1-weighted images at (**c**) 6 months and (**d**) > 2 years after completion of radiotherapy demonstrate a small pericavitary enhancing focus (arrow in **c**) with complete resolution on long-term follow-up; the dashed circle in **d** marks the prior lesion location

Onset is later than in IDH-wildtype glioblastoma but spans a wide window: reported median onset ranges from approximately 5 to 11 months, about two-thirds occur beyond 6 months, one-third within; and it should be considered throughout the first 2 years after radiotherapy [25, 26]. Lesions are typically small (less than 1 cm), nodular, and solitary or few, deep-seated or subependymal, and within the radiation field (Fig. 3) [26]; this may reflect the increased susceptibility of periventricular regions to treatment-related injury [27]. Perfusion usually shows no rCBV increase [26]. Separately, a non-enhancing pattern has also been described after PCV chemotherapy alone, with new or enlarging pericavitary white matter T2/FLAIR hyperintensity [28]. PsP is predominantly asymptomatic, resolves in about two-thirds (median duration approximately 8 months), and carries no adverse prognostic impact, supporting a conservative observation-based approach in clinically stable patients [25, 26].

**Fig. 4:**
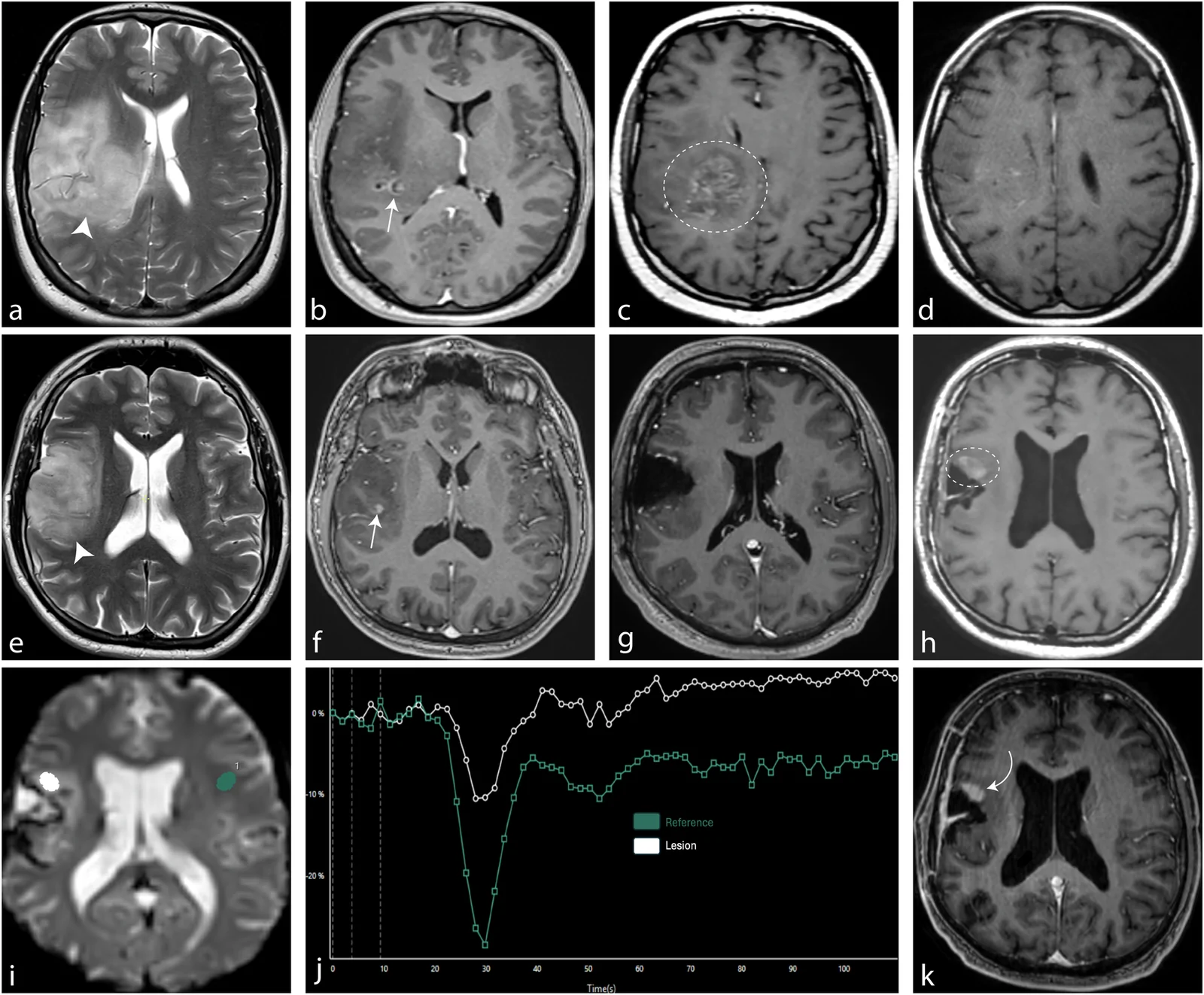
Pseudoprogression after chemoradiotherapy in IDH-mutant astrocytoma, CNS WHO grade 4. Two patients with IDH-mutant astrocytoma, CNS WHO grade 4, treated with radiotherapy and concurrent and adjuvant temozolomide. Case 1 (**a**–**d**). **a** Preoperative axial T2-weighted image showing a right hemispheric mass (arrowhead). **b** Preoperative axial post-contrast T1-weighted image showing minimal ring enhancement (arrow). **c** Axial post-contrast T1-weighted image 3–4 months after completion of radiotherapy, showing a clumpy, heterogeneous, Swiss-cheese-like enhancing focus at the tumour site (dashed circle). **d** Axial post-contrast T1-weighted follow-up shows almost total resolution of enhancement. Case 2 (**e**–**k**). **e** Preoperative axial T2-weighted image showing a right frontal mass (arrowhead). **f** Preoperative axial post-contrast T1-weighted image showing minimal enhancement (arrow). **g** Immediate postoperative axial post-contrast T1-weighted image showing the resection cavity without residual nodular enhancement. **h** Axial post-contrast T1-weighted image 2 months after completion of radiotherapy, showing new nodular enhancement at the anterior margin of the resection cavity (dashed oval). **i** Dynamic susceptibility contrast (DSC) perfusion source image showing the regions of interest used for analysis: the enhancing lesion (white) and contralateral normal-appearing white matter (teal, labelled 1). **j** Corresponding time–signal intensity curves, colour-coded as shown in the key within the image: the lesion curve shows a shallower signal drop with prompt return towards baseline compared with the reference curve. **k** Follow-up axial post-contrast T1-weighted image showing a mild reduction in size of the enhancing focus (curved arrow)

Grade 4 astrocytoma is a partial exception, and the evidence conflicts. In the POLA cohort, its frequency, timing and morphology did not differ from other IDH-mutant subtypes [26], whereas a large single-institution series reported the highest pseudoprogression incidence in this subtype (32%) and found that, uniquely, time of onset and radiographic features did not distinguish pseudoprogression from true progression [25]. Early confluent enhancement resembling pseudoprogression in IDH-wildtype glioblastoma may occur in some cases (Fig. 4).

#### Stereotactic radiosurgery

SRS has a limited role in diffuse gliomas because focal treatment does not address infiltrative disease. Reported uses include selected recurrent high- or low-grade gliomas, boost treatment to residual or postoperative disease, and selected brainstem gliomas; bevacizumab may be combined with SRS in recurrent glioblastoma to reduce radiation-related toxicity [29]. Prospective evidence for focal reirradiation remains limited, and SRS is reserved for small, well-defined lesions.

Because SRS is used mainly for brain metastases, its post-treatment changes—and broader radiation-related changes, which depend on dose, field, and timing rather than tumour type—are described in Section II.

### Systemic therapies

#### Targeted therapy

##### IDH inhibitor–related imaging changes.

IDH inhibitors, including ivosidenib and vorasidenib, suppress D-2-hydroxyglutarate production. A transient increase in normalised rCBV may occur within 3–6 weeks without a corresponding ADC change, potentially reflecting treatment-related effects. Imaging at 2–4 months may provide a more reliable assessment of response [30].

##### Anti-angiogenic drug–related imaging patterns: bevacizumab and regorafenib.

Bevacizumab reduces vascular permeability and vasogenic oedema, producing a rapid decrease in enhancement without equivalent tumour control—a pseudoresponse [5, 31, 32]. Regorafenib inhibits VEGF receptors and additional angiogenic, oncogenic, and stromal kinases, and its imaging effects overlap with but are not identical to those of bevacizumab [33].

**Fig. 5:**
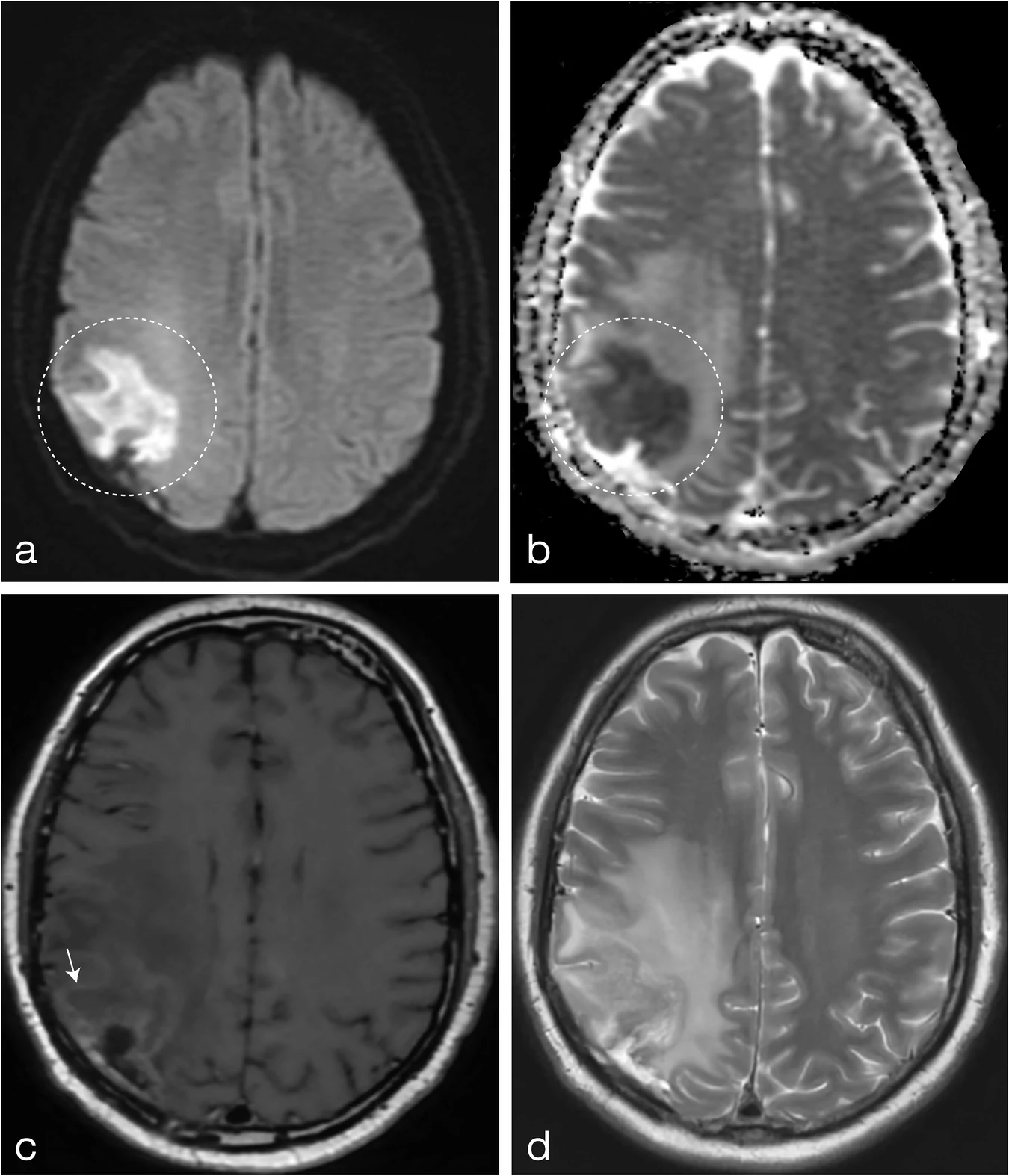
Bevacizumab-induced coagulative necrosis. A patient with a history of right-sided glioblastoma after surgical debulking, chemoradiotherapy and adjuvant temozolomide, with residual/recurrent disease; the patient is on bevacizumab. **a**, **b** Axial diffusion-weighted image (DWI) and apparent diffusion coefficient (ADC) map showing geographic focal markedly restricted diffusion abutting the anterior aspect of the resection cavity (white dashed circles). **c** Axial post-contrast T1-weighted image at the same level; the white arrow indicates the area corresponding to the diffusion abnormality, which shows no enhancement; note the peripheral thin intrinsic high T1 signal. **d** Axial T2-weighted image at the same level demonstrating corresponding heterogeneous T2 hyperintensity

Under bevacizumab, progression after pseudoresponse takes two forms: classical enhancing recurrence—the return or flare of measurably enhancing tumour—and T2-dominant progression, a predominantly non-enhancing expansion of T2/FLAIR signal, which is easily missed if enhancement alone is followed [31]. Under regorafenib, two patterns have been described: a conventional pattern with increasing enhancement, T2/FLAIR abnormality, and stable or increased rCBV; and a second pattern with reduced enhancement, increasing T2/FLAIR abnormality, marked diffusion restriction, low ADC, T2 hypointensity within the restricted component, an SWI hypointense rim, and reduced rCBV [33]. This second pattern may resemble bevacizumab-associated coagulative necrosis, typically sharply marginated diffusion restriction with very low ADC, low rCBV, and little enhancement, often in the periventricular white matter or corpus callosum (Fig. 5) [34, 35]. Stable lesions are associated with longer overall survival, whereas progressively enlarging ones carry worse outcomes, and they occur almost exclusively in MGMT-unmethylated tumours [34, 35]. Under regorafenib, however, histologically confirmed cases remain limited [36].

#### Immunotherapy-related imaging changes

Diffuse gliomas are immunologically cold tumours, and immune checkpoint inhibitor monotherapy has shown limited efficacy in glioblastoma [37]. Evidence for ICI-related pseudoprogression is likewise limited in diffuse gliomas compared with brain metastases [38]. Because most ICI imaging data derive from metastatic disease, these patterns are discussed in Section II.

Other immunotherapies aim in part to convert immunologically cold tumours into hot ones. Oncolytic viral therapy induces a proinflammatory microenvironment with cytotoxic T-cell infiltration [39]. Tumour vaccines similarly elicit inflammation at the tumour site [40]. MRI may show new or enlarging enhancement with perilesional T2/FLAIR hyperintensity, typically within 6 mo of starting treatment, later stabilising or regressing, consistent with immune-mediated pseudoprogression [40]. Because enhancement and FLAIR reflect only blood–brain barrier disruption and oedema, they cannot separate this from true progression; evidence remains largely trial-based, and treatment-specific morphological patterns are still incompletely characterised [39, 40].

Perfusion findings in immunotherapy-related pseudoprogression may differ from those of classic post-chemoradiation treatment effects. A recent study of ICI-treated brain metastases found relatively higher rCBV in immunotherapy-related pseudoprogression than in chemoradiation-related pseudoprogression or radiation necrosis, suggesting that conventional low-perfusion thresholds may not be directly transferable [41]. In glioblastoma treated with dendritic-cell vaccination, however, longitudinal change appeared more informative than a single measurement: increasing rCBV favoured true progression [42].

CAR-T therapy remains investigational in adult diffuse gliomas, including glioblastoma and H3 K27-altered diffuse midline glioma. Its main imaging relevance is tumour inflammation–associated neurotoxicity (TIAN), an on-target reaction caused by local immune activation at the CNS tumour site. TIAN has been observed in gliomas and primary CNS lymphoma, within days to weeks of CAR-T administration. Two patterns have been described: type 1, inflammatory oedema with raised intracranial pressure, which may manifest as peritumoural oedema, hydrocephalus, mass effect, or signs of herniation; and type 2, transient neurological dysfunction with minimal or no imaging correlate [43, 44]. These changes may mimic early tumour progression and should be interpreted with the treatment route, target antigen, timing, neurological status, and steroid or anti-inflammatory treatment history. TIAN should be distinguished from immune effector cell-associated neurotoxicity syndrome (ICANS), a systemic CAR-T complication that is not tumour-site-specific; imaging is often normal but may show cerebral oedema, diffusion restriction, or changes resembling posterior reversible encephalopathy syndrome [43].

### Locoregional techniques

#### Laser interstitial thermal therapy (LITT)–related imaging changes

LITT uses an MRI-guided, stereotactically placed laser probe to induce coagulative thermal necrosis. In neuro-oncology, it is applied chiefly to small, deep, or surgically inaccessible lesions, including recurrent gliomas and radiation necrosis.

**Fig. 6:**
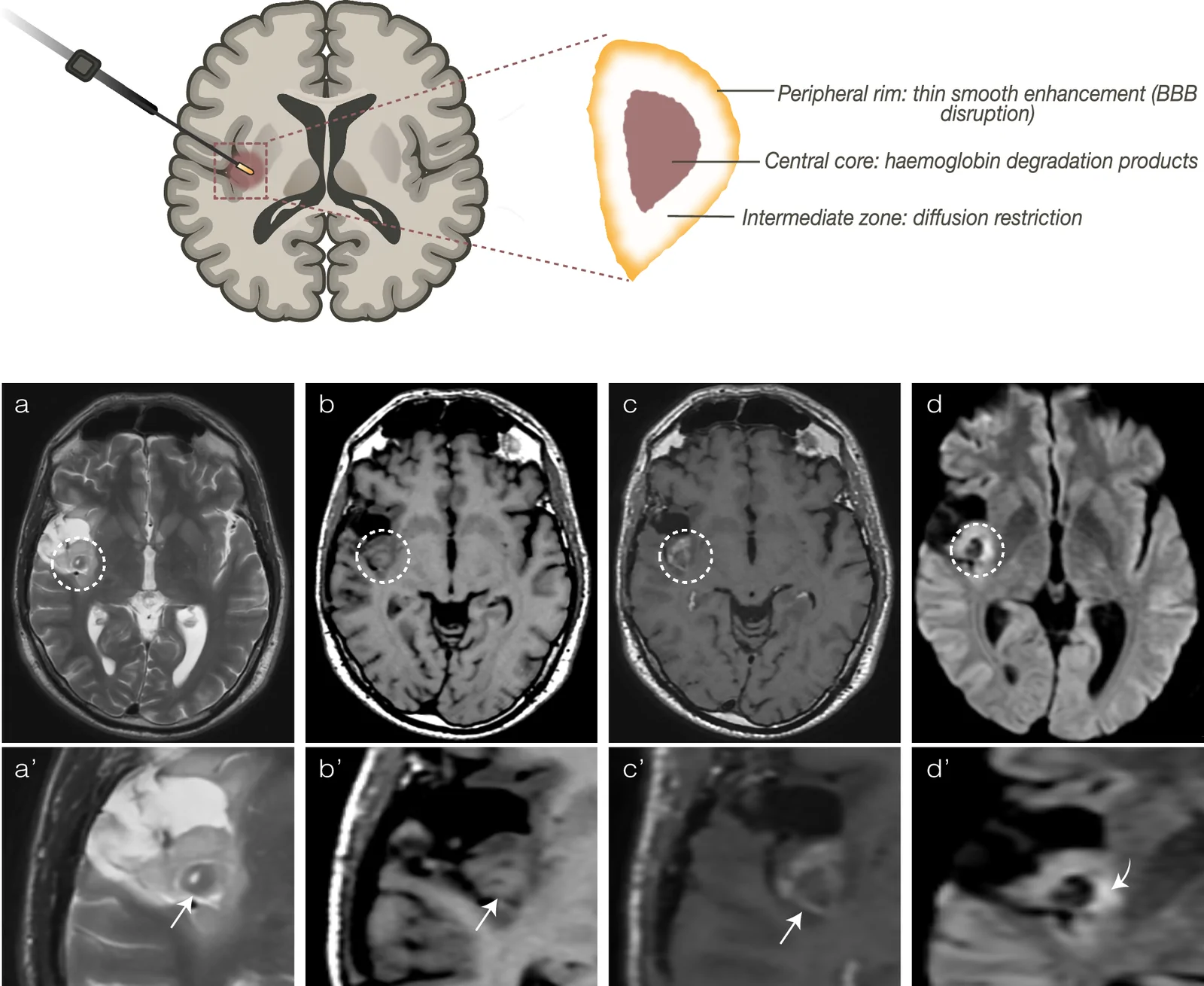
Concentric zonal architecture of the ablation area on early post-LITT MRI. Top: schematic of laser interstitial thermal therapy (LITT). The axial brain illustration (left) shows the laser applicator in position; the magnified inset (right) depicts the three concentric zones of an ablation cavity—central core (haemoglobin degradation products, T1 hyperintense), intermediate zone of diffusion restriction, and peripheral rim of thin smooth enhancement (blood–brain barrier disruption). Bottom: serial axial MRI in a case of recurrent high-grade astrocytoma, obtained 2 weeks after LITT. **a**–**d** Wide-view axial images showing the ablation zone in the right temporal lobe (dashed circle, all panels): **a** T2-weighted; **b** pre-contrast T1-weighted; **c** post-contrast T1-weighted; **d** diffusion-weighted (DWI). **a′**–**d′** Magnified views of the ablation cavity (arrow, all panels): **a′** low T2 signal in the central core; **b′** iso-T1 signal in the central core, consistent with haemoglobin degradation products; **c′** thin peripheral rim of enhancement representing the outer edge of thermal injury; **d′** rim of diffusion restriction at the intermediate zone

The ablation zone evolves through predictable phases (Fig. 6). Immediately after ablation, usually within 48 h, MRI shows a target-like lesion: a central T1-hyperintense zone of coagulative necrosis related to membrane disruption and haemoglobin breakdown products; a surrounding T1-hypointense to isointense zone of irreversible thermal injury and necrotising oedema, often with rim-like diffusion restriction; and a thin, avidly enhancing peripheral rim representing blood–brain barrier disruption and the outer margin of thermal injury [45–47]. Perilesional vasogenic oedema lies beyond this rim and is usually minimal at this stage. The enhancing rim approximates the extent of ablation; residual enhancing tumour beyond it suggests subtotal treatment [47].

During the subacute phase, rim enhancement and vasogenic oedema increase, the zonal pattern becomes less distinct, and transient lesion enlargement may occur, potentially mimicking progression [45]. Over subsequent months, the ablation zone involutes centripetally: oedema resolves, the enhancing rim thins, and in the chronic phase, often after 100 days, gliosis predominates, with minimal residual enhancement and possible cavity formation [45]. Expected treatment effect is suggested by serial contraction and fading enhancement, whereas new or enlarging nodular enhancement, particularly if accompanied by rising perfusion, should raise concern for residual or recurrent tumour.

#### Tumour-treating fields

Tumour-treating fields (TTFields) are wearable scalp arrays delivering low-intensity alternating electric fields that disrupt mitotic spindle formation, used only in glioblastoma, with adjuvant temozolomide in newly diagnosed disease, or as monotherapy at recurrence [48, 49]. Apparent tumour growth may appear within the first months, although this is difficult to separate from pseudoprogression related to concurrent chemoradiotherapy; less commonly, it has been reported beyond the usual post-chemoradiation window [49, 50]. TTFields may also transiently increase blood–brain barrier permeability, raising the Ktrans on DCE perfusion and rCBV on DSC perfusion across the 2–6 mo on-treatment interval—the opposite of the decline seen with temozolomide alone [51].

#### Imaging changes related to blood–brain barrier modulation for drug delivery

Focused ultrasound with intravenous microbubbles transiently and reversibly opens the blood–brain barrier to enhance systemic drug delivery and remains investigational in glioblastoma and brain metastases. Successful opening appears as a new focal enhancement within the sonicated target, best seen within approximately 1 h, often as multiple punctate or linear foci reflecting the perivascular distribution. SWI may show corresponding punctate low-signal foci without overt haemorrhage [52, 53].

## Section II: Treatment-related imaging changes in brain metastases

### Postoperative imaging changes

Expected postoperative changes are not tumour-specific and are described in Section I.

### Imaging changes after radiotherapy-based treatment

#### MRI patterns after stereotactic radiosurgery (SRS)

Stereotactic radiosurgery delivers high-dose focal irradiation to a defined intracranial target and is preferred over whole-brain radiotherapy for limited brain metastases [54]. The main interpretive challenge is distinguishing tumour progression from radiation-related change, which ranges from transient post-SRS enlargement that subsequently stabilises or regresses without treatment change, to more persistent radiation necrosis. This transient enlargement is often termed pseudoprogression in the SRS literature. Radiation-related change most often appears between approximately 6 weeks and 15 months after treatment, although necrosis may occur later [54, 55].

**Fig. 7:**
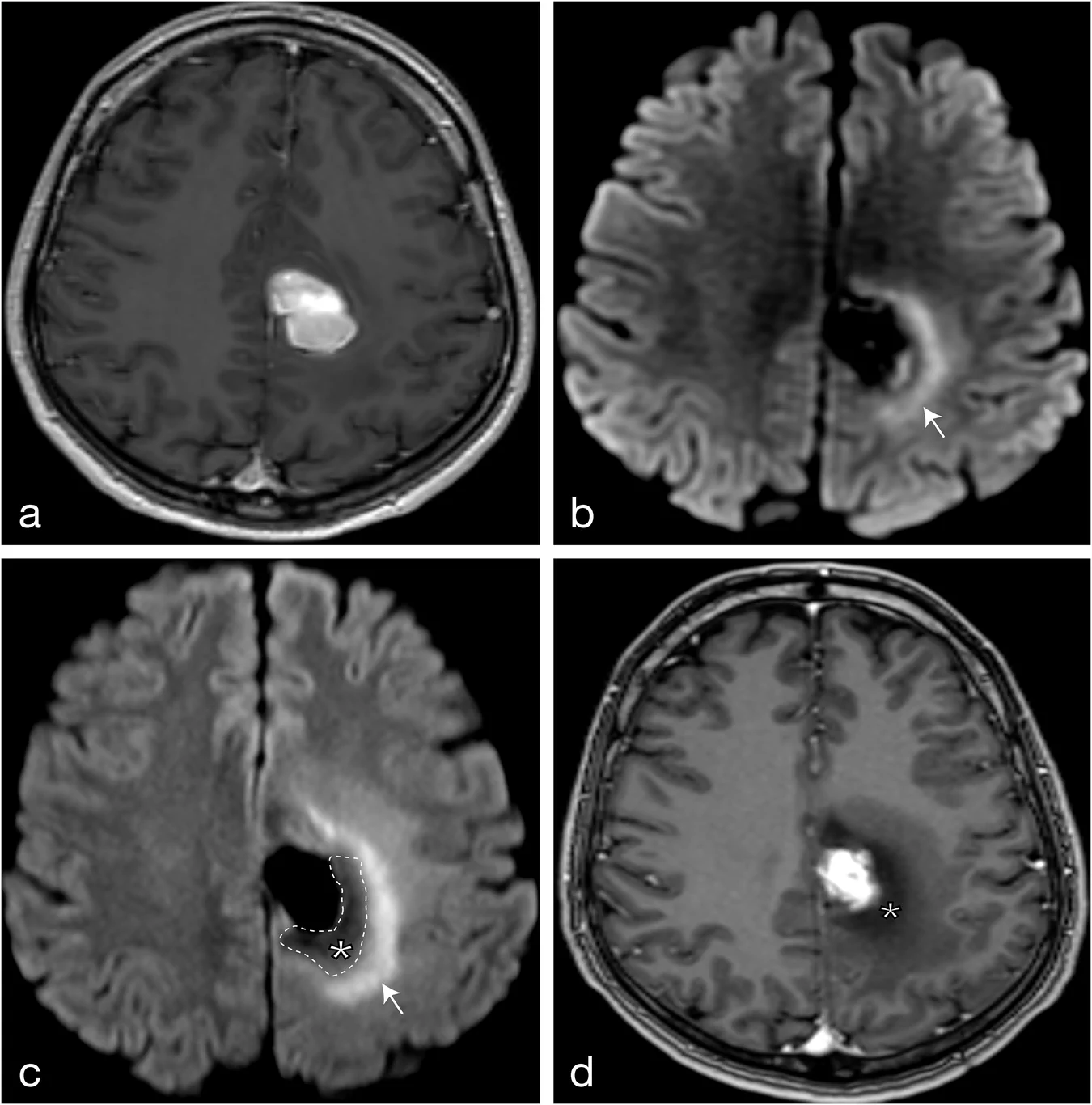
Radiation-induced white matter injury after stereotactic radiosurgery (SRS) for a parafalcine brain metastasis. **a** Pre-SRS axial post-contrast T1-weighted image showing the target metastasis. **b** Axial diffusion-weighted image at 12 months after SRS demonstrating a curvilinear band of high diffusion signal in the adjacent white matter (arrow), suggestive of demyelination. **c** Axial diffusion-weighted image at 32 months after SRS showing two co-existing zones: a peripheral band of high diffusion signal with directional progression along the white matter compared to panel b (arrow), suggestive of demyelination, and a central zone of low diffusion signal (dashed outline and asterisk), likely representing established gliosis. **d** Axial T1-weighted image at 32 months showing T1 hypointensity at the same site as the low-DWI zone in c (asterisk), in keeping with chronic gliosis

Most metastases shrink early after stereotactic radiosurgery, with the greatest volume reduction during the first 1–3 months. Perilesional oedema follows a similar course, decreasing most markedly within 1–3 months and continuing to resolve up to approximately 8 months [56]. Against this background, transient enlargement occurs in approximately 25–33% of treated lesions, typically beginning at 1–3 months, peaking around 3–6 months, and occasionally persisting or fluctuating up to approximately 15 months [56, 57]. Interval growth alone therefore lacks specificity for true progression [54]. Lesions with poor baseline peritumoural perfusion and vessel pruning may be more susceptible to pseudoprogression [58]. Adjacent white matter may undergo delayed radiation-related demyelination followed by gliosis; on MRI, this may appear as a diffusion abnormality that later evolves into T1 hypointensity and focal volume loss (Fig. 7).

**Distinguishing radiation necrosis from metastasis progression**: Radiation necrosis and progressive metastasis may both appear as peripherally enhancing lesions with a central non-enhancing component. The discriminating features are summarised in Table 2.

**Table 2:** Features favouring radiation necrosis versus metastasis progression after SRS [73, 102–109]

Feature	Favours radiation necrosis	Favours metastasis progression
Conventional MRI		
Enhancement morphology	Thin, irregular peripheral enhancement · no discrete solid nodule [54, 55]	Thick or nodular enhancement · discrete solid component [55]
Margins and location	Crosses anatomical boundaries · multiple clustered foci within field · gyriform margins in juxtacortical lesions · interval shape change [55]	Mass-like margins conforming to the original lesion [55]
SWI	Multifocal punctate susceptibility foci, reflecting microvascular injury [55, 102]	Haemorrhagic metastases and intratumoural haemorrhage may mimic [55, 102]
T1/T2 mismatch	Indistinct T2 margin relative to the enhancing T1 abnormality [102]	T2 margin matching the enhancing T1 abnormality [102]
Diffusion		
DWI/ADC pattern^a^	Central restriction within a ring-enhancing lesion [54, 103]	Peripheral-rim or solid-component restriction [54, 103]
ADC evolution^b^	Three-layer pattern: high central · low intermediate · higher peripheral ADC [104] Rising mean ADC over time [56, 105]	Low ADC in the enhancing or solid component [104, 105] Falling mean ADC [56, 105]
Perfusion and metabolism		
rCBV (DSC)	Reduced [54, 55]	Elevated [54, 55]
MRS	Low choline · low choline/NAA · early choline reduction in responders [106]	Rising choline · falling NAA · high choline/NAA [106]
Amino-acid PET	Low tumour-to-brain uptake [54, 73]	High tumour-to-brain uptake [54, 73]
Adjunct contrast techniques^c^		
Delayed post-contrast imaging	Persistent delayed enhancement [107]	Contrast washout [107]
Gd-enhanced T2-FLAIR	Higher T2-FLAIR signal [108]	Lower T2-FLAIR signal [108]
Clinical and temporal modifiers		
Patient/treatment factors	Older age · longer interval from SRS · larger irradiated volume [109]	Shorter interval · progressive clinical decline [109]

^a^ Distribution of diffusion restriction is more discriminating than its presence alone

^b^ Combined volume–ADC diffusion indices have been proposed for early discrimination [105]

^c^ Adjunct contrast techniques have limited validation and should be interpreted as supportive rather than definitive [107, 108]

##### Special situations

**Fig. 8:**
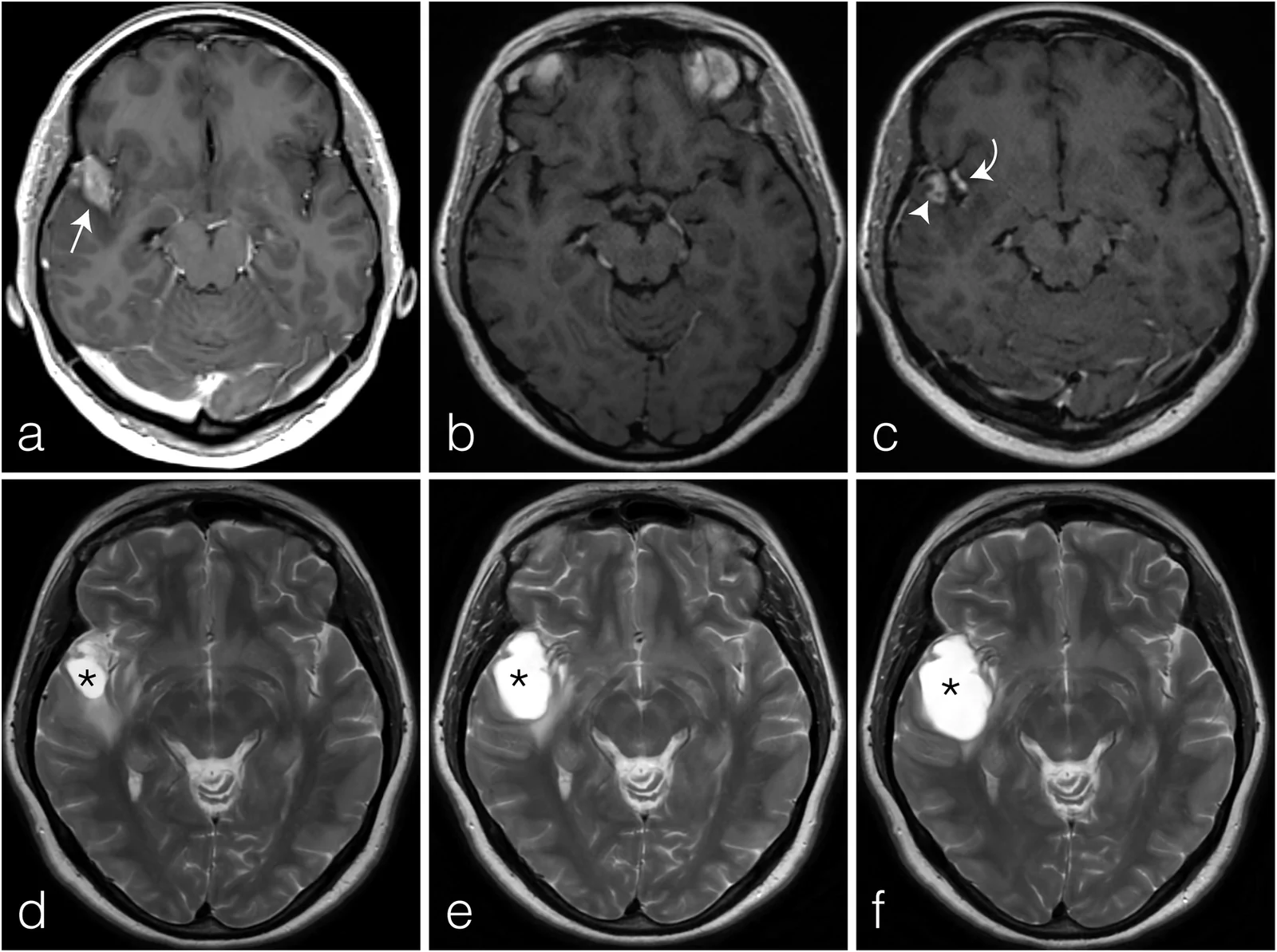
Cystic formation following stereotactic radiosurgery (SRS) of a brain metastasis. Serial MRI in a patient with breast cancer brain metastasis treated with SRS. **a** Axial post-contrast T1-weighted image at presentation: enhancing metastatic lesion in the right anterior temporal lobe (arrow). **b** Four months after SRS, axial post-contrast T1-weighted image: post-treatment response, with no appreciable residual lesion or enhancement at the treated site. **c** Two years after SRS, axial post-contrast T1-weighted image: small focus of enhancement at the original site (arrowhead) and a second focus on the opposing cortical bank across the adjacent sulcus (curved arrow). **d**–**f** Axial T2-weighted images obtained 3 years, 3 years 6 months, and 4 years after SRS, respectively: resolution of the enhancement and progressive cystic change at the treated site (asterisk), enlarging over time without solid or enhancing components

**Fig. 9:**
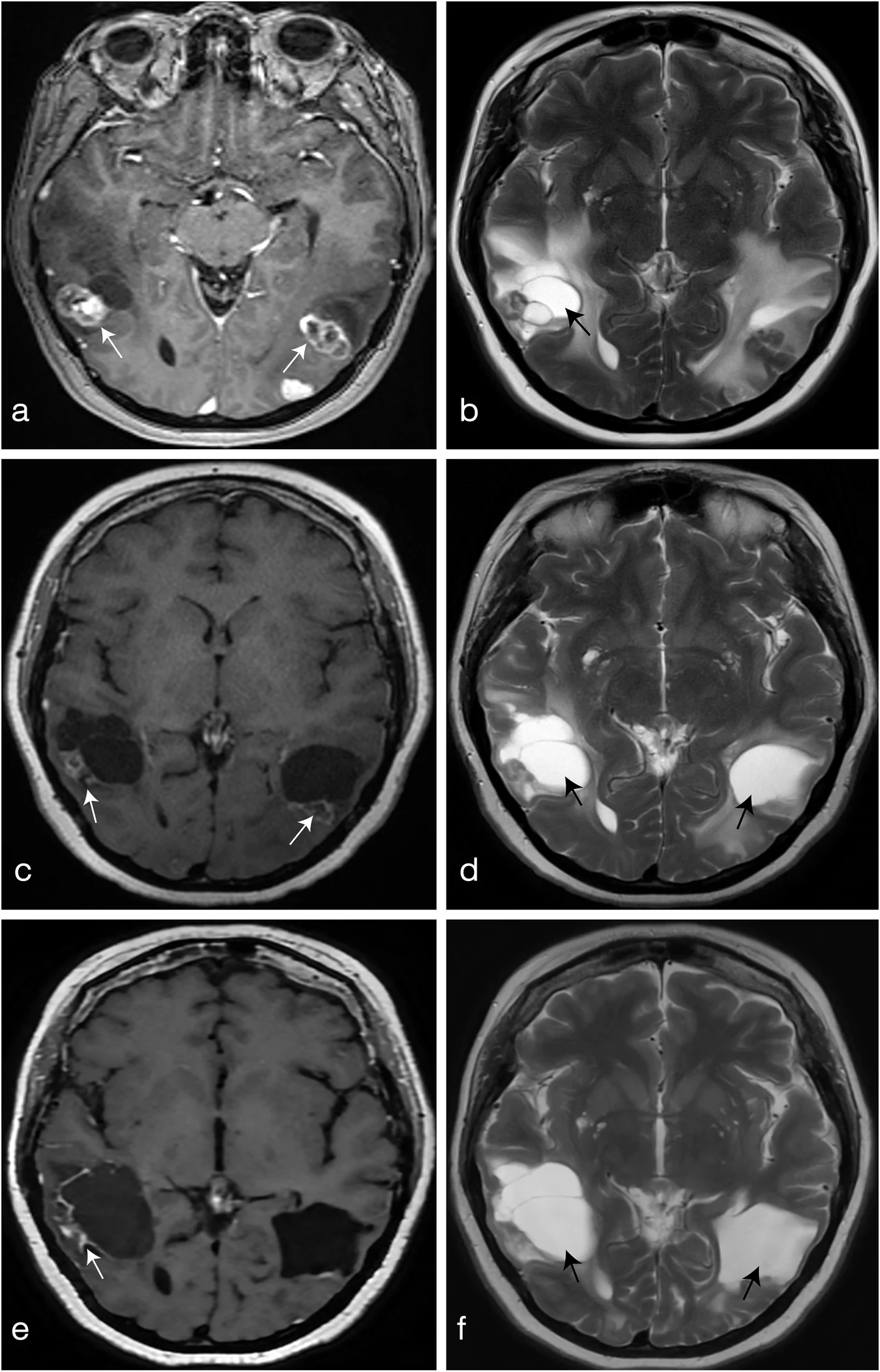
Progressive enlargement of the cystic component of brain metastases after stereotactic radiosurgery. A patient with metastatic breast cancer treated with stereotactic radiosurgery (SRS) for bilateral cerebral metastases. **a**, **b** Pre-SRS axial post-contrast T1-weighted (**a**) and T2-weighted (**b**) images showing bilateral enhancing solid-cystic cerebral metastases; white arrows in **a** mark the enhancing solid components, black arrow in **b** indicates the cystic component of the right cerebral lesion. **c**, **d** Three-month post-SRS images at the same level showing decreased size of the enhancing solid components (white arrows in **c**) and interval enlargement of the cystic components (black arrows in **d**). **e**, **f** Twelve-month post-SRS images showing further reduction/resolution of the enhancing solid components and progressive enlargement of the cystic components (white arrow in **e**; black arrows in **f**)

A progressively enlarging non-enhancing cavity may develop as a delayed complication (Fig. 8); such cavities rarely regress spontaneously, often require medical or surgical intervention, and are markedly more frequent after repeating stereotactic radiosurgery [59]. Pre-existing cystic components may also enlarge during treatment (Fig. 9) [60]. Similar transient post-SRS enlargement is recognised in benign tumours, particularly vestibular schwannomas, but this is outside the main scope of this section [61, 62].

#### Whole-brain radiotherapy

Whole-brain radiotherapy is now used selectively for brain metastases, particularly for multiple or disseminated intracranial disease, leptomeningeal involvement, or when focal therapy is unsuitable.

**Changes after radiation therapy**: Although discussed here in the context of whole-brain radiotherapy, these changes are not specific to it. Any radiation-based treatment, including focal radiotherapy and stereotactic radiosurgery for either glioblastoma or metastases, can produce a similar spectrum of effects. These are determined mainly by dose, fractionation, treated volume, and time since irradiation rather than by tumour type alone; interpretation therefore depends on knowing the field, dose, and timing [43, 63].

Radiation necrosis is addressed in the preceding sections; this section covers the broader spectrum of late and chronic radiation-related effects.

##### White matter injury

Radiation-induced leukoencephalopathy is a common delayed complication, occurring months to years after irradiation, with risk increasing with dose, treated volume, and concurrent methotrexate. MRI shows symmetric periventricular T2/FLAIR hyperintensity without enhancement or mass effect, which may progress to diffuse white matter involvement and cerebral atrophy, typically sparing the subcortical U-fibres, corpus callosum, and cortex [43, 63].

##### Vascular injury

**Fig. 10:**
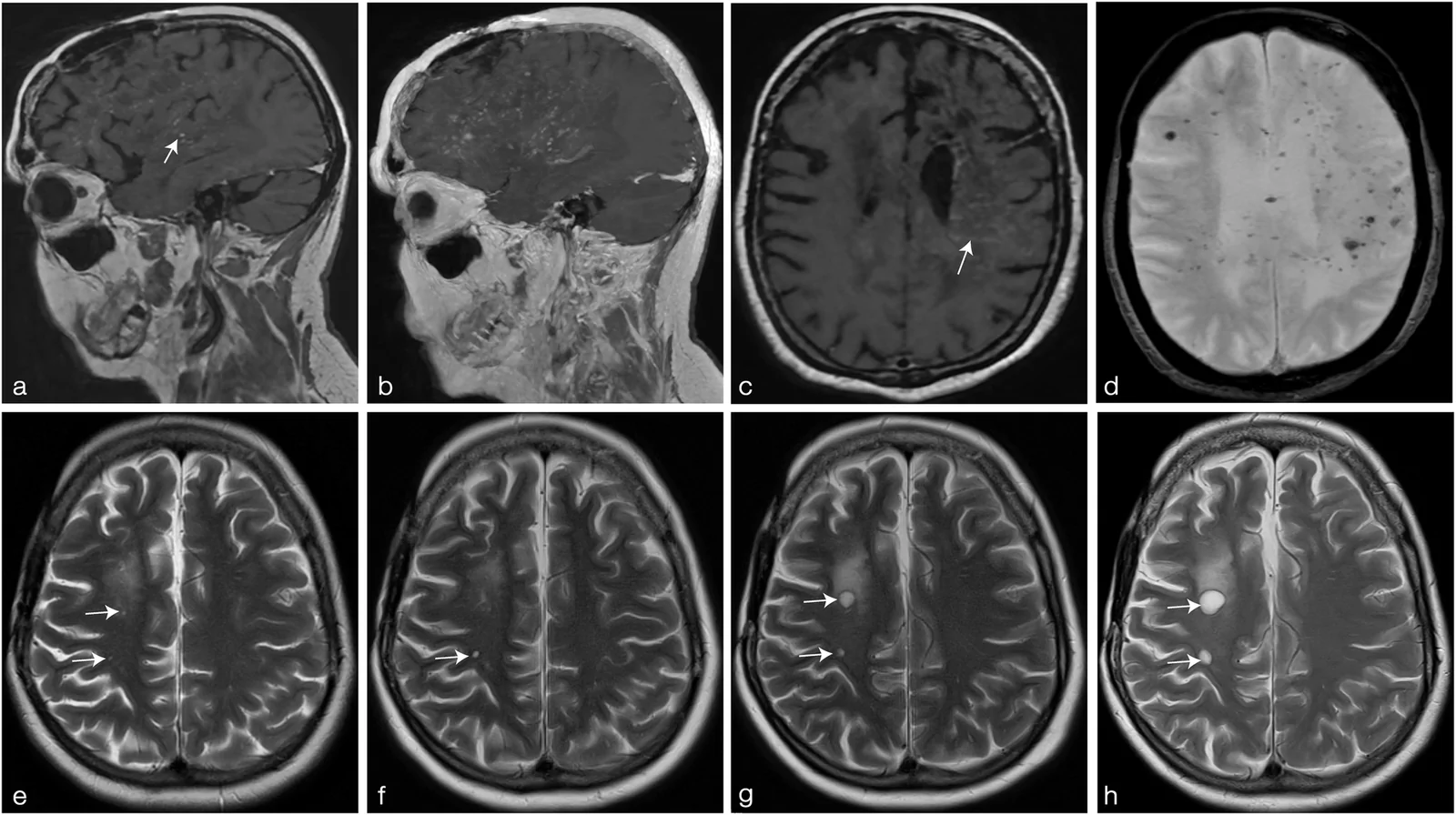
Spectrum of late-delayed perivascular changes after cranial radiotherapy. Two long-term survivors of cranial radiotherapy demonstrating distinct but related manifestations of late-delayed radiation injury localised to the perivascular (Virchow–Robin) space. Case 1 (**a**–**d**). A long-term survivor of cranial radiotherapy. **a** Sagittal post-contrast T1-weighted image showing linear and punctate enhancing foci within the irradiated field (white arrow). **b** Sagittal maximum-intensity-projection (MIP) reconstruction demonstrating the enhancing foci arranged in a branching, linear perivascular configuration. **c** Axial post-contrast T1-weighted image confirming the enhancing foci oriented perpendicular to the ventricular margin, in keeping with a perivascular distribution along the deep medullary vessels (white arrow). **d** Axial gradient-echo T2*-weighted image at the same level demonstrating multiple low-signal foci in keeping with susceptibility-related signal loss from microhaemorrhage at the same region. Case 2 (**e**–**h**). Serial axial T2-weighted images from a long-term survivor of cranial radiotherapy at sequential follow-up timepoints (**e** before radiotherapy), demonstrating progressive enlargement of focal perivascular spaces within the irradiated parasagittal white matter (white arrows) in the right cerebral hemisphere. The lesions enlarge over time without contrast enhancement, in keeping with focal Virchow–Robin space expansion rather than tumour recurrence or radiation necrosis. Both patterns are centred on the perivascular spaces, which form part of the brain’s glymphatic system; their involvement reflects the susceptibility of this compartment to late radiation injury

Radiation produces a spectrum of vascular injury through endothelial damage. Large-vessel vasculopathy may involve the circle of Willis or proximal carotid arteries, showing concentric wall thickening and enhancement, with potential progression to stenosis or moyamoya-like collateralisation. Radiation-induced aneurysms, most commonly involving the internal carotid artery, may carry an increased rupture risk and warrant surveillance [43, 63]. Microvascular injury includes radiation-induced cavernous malformations, mineralising microangiopathy, and perivascular enhancement, thought to reflect small-vessel microvasculitis and possible glymphatic dysfunction. This may manifest as small, 10 mm or smaller, punctate, oval, or rod-shaped enhancing foci within the radiation field, typically emerging at least 24 months after high-dose treatment of 54 Gy or higher, frequently associated with ipsilateral Virchow–Robin space dilatation (Fig. 10), and in some cases evolving into T2*WI-hypointense microbleeds [64–66]. Intravascular papillary endothelial hyperplasia is a rare benign vascular proliferation that may mimic malignancy [43].

##### SMART syndrome

Stroke-like migraine attacks after radiation therapy syndrome typically occur many years after treatment and present with headache, focal deficits, and seizures [67, 68]. MRI shows transient unilateral gyriform cortical T2/FLAIR hyperintensity and enhancement confined to the radiation field, developing over days and resolving over 2–5 weeks [67]. Increased CBV or CBF on perfusion, which may subsequently normalise, may help distinguish this syndrome from ischaemia or recurrence [68, 69]. Two related entities probably sit within the same spectrum [70]: peri-ictal pseudoprogression is seizure-driven, with focal cortical or leptomeningeal enhancement that resolves once seizures are controlled [71]; acute late-onset encephalopathy after radiation therapy typically follows whole-brain radiotherapy and is more severe, with diffuse encephalopathy and multifocal involvement [70].

##### Other late effects

Radiation-induced cranial neuropathy appears as smooth nerve thickening and enhancement, most commonly involving the optic pathways, and may progress to atrophy. Hypopituitarism manifests as progressive pituitary atrophy. There is also a long-term risk of secondary neoplasms, most commonly meningiomas and less frequently gliomas or sarcomas [43].

### Systemic therapies

#### Immunotherapy-related imaging changes

Unlike the immunologically cold gliomas discussed in Section I, many brain metastases, particularly melanoma and non-small cell lung cancer, retain a hot immunogenic profile. This underlies both higher response rates to immune checkpoint inhibitors and a higher incidence of immune-related imaging change [37]. Most intracranial neuroimaging experience therefore derives from ICI-treated cohorts, and ICI-related change falls into two overlapping categories: atypical response patterns and intracranial immune-related adverse events (irAEs) [72].

##### Atypical response patterns.

Immunotherapy-induced pseudoprogression is transient radiological worsening, with new or increased enhancement and/or T2/FLAIR expansion that later stabilises or regresses, usually within 6 months and rarely later. Unlike chemoradiotherapy-related pseudoprogression, it reflects immune-mediated inflammatory infiltration rather than vascular injury [73]. Reported rates in immunogenic metastases, mainly melanoma and non-small cell lung cancer, are approximately 10%, whereas evidence in glioblastoma is limited [38, 74].

Radiographic characterisation remains limited. Three patterns have been described: new or enlarging enhancement at known tumour sites, along cranial nerves, or at distant sites outside prior radiation fields, possibly reflecting unmasking of occult disease; less commonly, T2/FLAIR-predominant change; and vasculitis-like perivascular enhancement with multifocal diffusion restriction, overlapping with immune-related cerebral vasculitis [75]. Immunotherapy-induced pseudoprogression may show higher rCBV than chemoradiotherapy-related pseudoprogression or radiation necrosis, limiting conventional rCBV thresholds [41]. Neuroinflammation-targeted techniques, including USPIO-enhanced MRI and TSPO-PET, may improve specificity [76].

Other atypical patterns include hyperprogression, defined as accelerated growth after immune checkpoint inhibitor initiation on sequential imaging; dissociated response, with concurrent responding and progressing lesions; abscopal response, with regression of distant lesions after local treatment through systemic immune activation; and durable response, with sustained control after treatment discontinuation [37, 72].

##### Intracranial immune-related adverse events

Hypophysitis is the most frequent intracranial immune-related adverse event, presenting as transient pituitary enlargement with homogeneous enhancement and mild stalk thickening in a normal-sized sella. A characteristic feature, particularly with ipilimumab, is geographic anterior lobe hypoenhancement with T2 hypointensity, reflecting fibrosis; baseline comparison is essential for subtle cases [77, 78]. Other intracranial immune-related adverse events include meningoencephalitis, seen as limbic encephalitis or diffuse leptomeningeal enhancement and sometimes MRI-occult; cranial neuritis with smooth nerve enhancement; and cerebral vasculitis with multifocal diffusion-restricted infarcts [77].

#### Other systemic and combined therapies

Brain metastases are increasingly treated with molecularly targeted agents, including EGFR, ALK, BRAF/MEK, and HER2 inhibitors. Unlike immunotherapy, these have not been linked to consistent intracranial pseudophenomena, although dissociated responses may be encountered [73]. The clearest exception is VEGF-pathway inhibition, best established with bevacizumab, which can rapidly reduce enhancement and oedema; in brain metastases, this is used mainly for symptomatic radiation necrosis. Conventional chemotherapy-related CNS toxicity is outside the scope of this review.

**Fig. 11:**
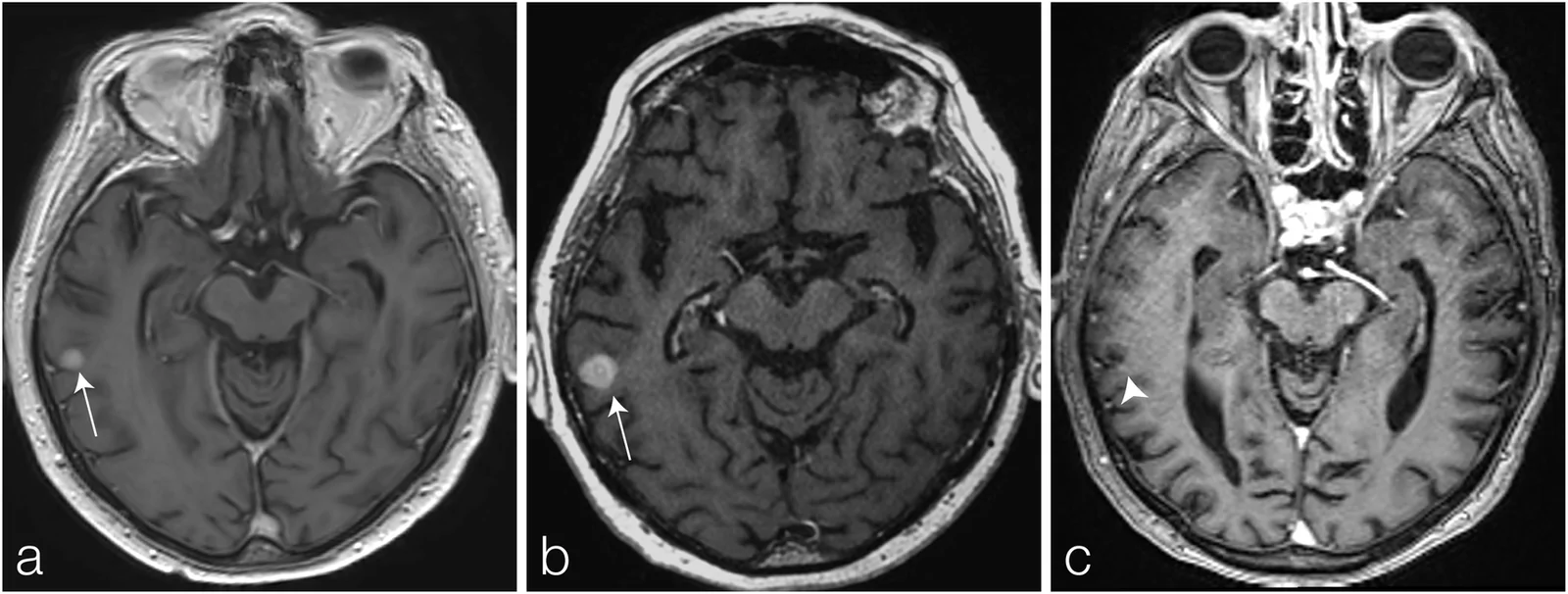
Brain metastasis treated with stereotactic radiosurgery (SRS) and immune checkpoint inhibitor therapy. **a** Pretreatment axial post-contrast T1-weighted image (arrow). **b** Follow-up at 4 months demonstrating transient enlargement of the treated lesion (arrow). **c** Follow-up at 8 months showing reduction in size (arrowhead). The findings are consistent with pseudoprogression in the setting of combined therapy; SRS-related and immune-related contributions overlap and cannot be reliably distinguished on imaging

Combining stereotactic radiosurgery with immune checkpoint inhibitors may increase both antitumour effect and radiation-necrosis risk, whereas the risk with targeted-therapy combinations is less consistent [73]. The main interpretive challenge is lesion worsening after both radiosurgery and immunotherapy, where the differential includes radiation necrosis, immunotherapy-induced pseudoprogression, and true tumour progression (Fig. 11) [79].

### Locoregional techniques

LITT is used for progressive lesions after stereotactic radiosurgery, whether recurrent metastatic disease or radiation necrosis [80]. Focused ultrasound blood–brain barrier opening has been investigated for drug delivery in brain metastases [53]. Both show the same imaging changes described in Section I.

## Conclusion and framework

**Fig. 12:**
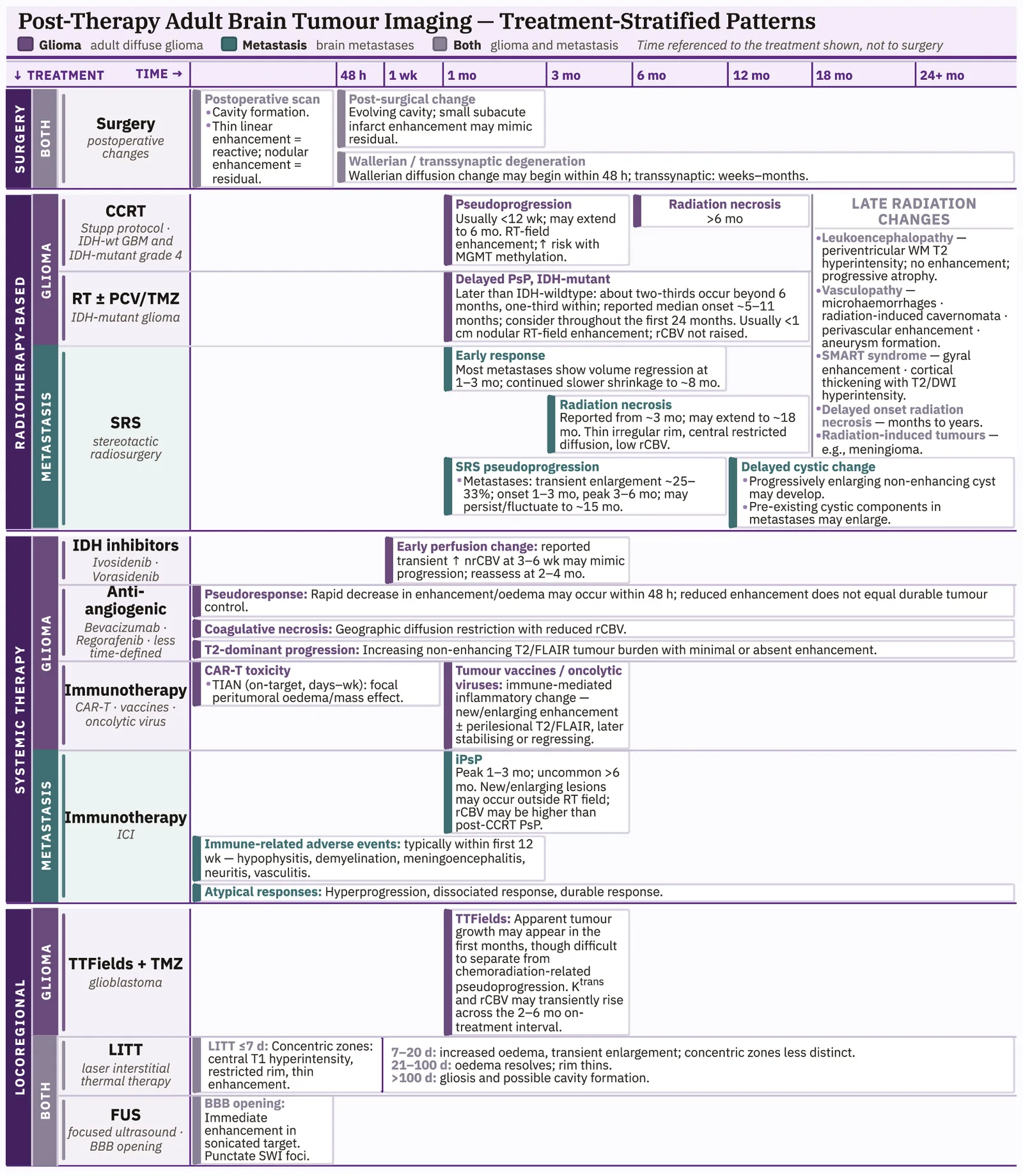
Treatment-specific patterns in post-therapy brain tumour imaging. Each row presents a treatment category and its characteristic post-treatment imaging changes, positioned along the typical time window in which they occur. The Late Radiation Changes panel applies across the three radiotherapy-based rows (CCRT, RT ± PCV/TMZ, SRS). Time is referenced to the treatment shown in that row, not to surgery. An interactive version is available as Additional File 1. BBB, blood–brain barrier; CAR-T, chimeric antigen receptor T-cell; CCRT, concurrent chemoradiotherapy; DWI, diffusion-weighted imaging; FLAIR, fluid-attenuated inversion recovery; FUS, focused ultrasound; ICI, immune checkpoint inhibitor; IDH, isocitrate dehydrogenase; iPsP, immunotherapy-induced pseudoprogression; K^trans^, volume transfer constant; LITT, laser interstitial thermal therapy; MGMT, O⁶-methylguanine-DNA methyltransferase; PCV, procarbazine, lomustine, and vincristine; PsP, pseudoprogression; rCBV, (nrCBV) (normalised) relative cerebral blood volume; RT, radiotherapy; SMART, stroke-like migraine attacks after radiation therapy; SRS, stereotactic radiosurgery; SWI, susceptibility-weighted imaging; TIAN, tumour inflammation–associated neurotoxicity; TMZ, temozolomide; TTFields, tumour-treating fields; WM, white matter

For radiologists, post-treatment brain tumour imaging is often a problem of context. The challenge is determining whether the findings represent an expected treatment effect or pseudo-phenomenon, or tumour progression. Figure 12 provides a treatment- and time-based overview of the major imaging patterns discussed in this review; an interactive version is provided as Additional File 1.

Pseudoprogression illustrates this principle. It is a conceptual, treatment-specific response pattern rather than a single imaging entity. Its likelihood, timing, and radiologic features vary according to tumour type and therapy. Table 3 compares the principal contexts in which the term is most used: pseudoprogression after concurrent chemoradiotherapy in IDH-wildtype glioblastoma, delayed pseudoprogression after radiotherapy in IDH-mutant glioma, and ICI-related pseudoprogression. It has also been applied to transient enlargement after SRS and to the inflammatory changes described in glioma trials of tumour vaccines and oncolytic viral therapy.

**Table 3:** Treatment-specific contexts of pseudoprogression

Feature	IDH-wildtype glioblastoma: post-chemoradiotherapy pseudoprogression	IDH-mutant glioma: delayed pseudoprogression	Brain metastases: Immunotherapy (ICI)–induced pseudoprogression
Frequency	Approximately 30% overall; more common with MGMT-promoter methylation.	Approximately 16–19%.	Approximately 9–10% at the lesion level.
Typical timing	Usually within 12 weeks after chemoradiotherapy; may extend to 3–6 months.	Later than IDH-wildtype but wide: median onset ~5–11 months; about two-thirds occur beyond 6 months, one-third within; consider throughout the first 24 months.	Usually within 2–3 months of treatment initiation and within 6 months.
Typical MRI pattern	New or enlarging, often confluent enhancement within the radiation field. rCBV is often lower than in progression, but overlap occurs, and recurrence within irradiated tissue may also show low rCBV.	Usually small (< 1 cm), nodular; solitary or few; deep-seated or subependymal; within the radiation field. rCBV usually not increased.	Enlargement or new enhancement of treated metastases; changes may occur outside the prior radiation field. rCBV may be relatively higher than in conventional treatment effect; thresholds remain unvalidated.
Associated treatment	Radiotherapy with concurrent and adjuvant TMZ.	Radiotherapy with or without PCV or TMZ.	ICI for systemic malignancies with brain metastases.

*ICI* immune checkpoint inhibitor, *IDH* isocitrate dehydrogenase, *MGMT* O⁶-methylguanine-DNA methyltransferase, *PCV* procarbazine, lomustine, and vincristine, *rCBV* relative cerebral blood volume, *TMZ* temozolomide

Interpreting these findings therefore depends less on any single sign than on placing the abnormality in its treatment and temporal context. Table 4 sets out a systematic approach on this basis. Figure 12 summarises the expected imaging patterns by treatment category and time from therapy.

**Table 4:** Practical framework for interpreting post-treatment brain tumour MRI

Step	Practical considerations
1. Treatment and baseline	Establish the tumour type and molecular status, the treatments given, and their sequence. Select the baseline that matches the treatment in question: early postoperative MRI for residual tumour, post-radiotherapy MRI in newly diagnosed glioma, or the pretreatment study for radiosurgery, systemic and ablative therapies.
2. Location and timing	Relation to the treated site: resection margin, radiation field or SRS target, ablation zone, or remote. Compare the interval since treatment with the expected window for that treatment (Fig. 12).
3. Pattern and interval change	Morphology, and whether the abnormality is enlarging, stable, or regressing.
4. Advanced imaging	Interpret perfusion, spectroscopy, diffusion, and amino-acid PET in treatment context; allow for artefact, threshold variability, anti-angiogenic and immunotherapy effects, mixed tumour and treatment effect, and prior radiation.
5. Impression	Treatment effect favoured, indeterminate or mixed, or progression favoured.

The framework is intended as a structured approach to interpretation rather than a diagnostic algorithm

*MRI* magnetic resonance imaging, *PET* positron emission tomography, *SRS* stereotactic radiosurgery

No single time point, imaging sign, or metabolic parameter is diagnostic in isolation, mixed tumour and treatment effect are common, and multidisciplinary review remains essential.

## Supplementary information

ELECTRONIC SUPPLEMENTARY MATERIAL

Additional_fileR1_1

## Data Availability

No datasets were generated or analysed during the current study.

## Abbreviations

ADC: Apparent diffusion coefficient
BBB: Blood–brain barrier
CAR-T: Chimeric antigen receptor T-cell
CNS: Central nervous system
DCE: Dynamic contrast-enhanced
DSC: Dynamic susceptibility contrast
DWI: Diffusion-weighted imaging
FLAIR: Fluid-attenuated inversion recovery
ICI: Immune checkpoint inhibitor
IDH: Isocitrate dehydrogenase
LITT: Laser interstitial thermal therapy
MGMT: O⁶-Methylguanine-DNA methyltransferase
PCV: Procarbazine, lomustine and vincristine
PET: Positron emission tomography
PsP: Pseudoprogression
RANO: Response Assessment in Neuro-Oncology
rCBV: Relative cerebral blood volume
SMART: Stroke-like migraine attacks after radiation therapy
SRS: Stereotactic radiosurgery
SWI: Susceptibility-weighted imaging
TIAN: Tumour inflammation–associated neurotoxicity
TMZ: Temozolomide
VEGF: Vascular endothelial growth factor

## References

[1] Scharl S, Kirstein A, Kessel KA et al (2019) Cavity volume changes after surgery of a brain metastasis—consequences for stereotactic radiation therapy. Strahlenther Onkol 195:207–217. 10.1007/s00066-018-1387-y 30386864

[2] Niyazi M, Andratschke N, Bendszus M et al (2023) ESTRO-EANO guideline on target delineation and radiotherapy details for glioblastoma. Radiother Oncol 184:109663. 10.1016/j.radonc.2023.109663 37059335

[3] Karschnia P, Young JS, Dono A et al (2023) Prognostic validation of a new classification system for extent of resection in glioblastoma: a report of the RANO resect group. Neuro Oncol 25:940–954. 10.1093/neuonc/noac193 PMC10158281 35961053

[4] Wen PY, van den Bent M, Youssef G et al (2023) RANO 2.0: update to the response assessment in neuro-oncology criteria for high- and low-grade gliomas in adults. J Clin Oncol 41:5187–5199. 10.1200/jco.23.01059 PMC10860967 37774317

[5] Martucci M, Russo R, Giordano C et al (2023) Advanced magnetic resonance imaging in the evaluation of treated glioblastoma: a pictorial essay. Cancers (Basel) 15:3790. 10.3390/cancers15153790 PMC10417432 37568606

[6] Bette S, Gempt J, Huber T et al (2016) Patterns and time dependence of unspecific enhancement in postoperative magnetic resonance imaging after glioblastoma resection. World Neurosurg 90:440–447. 10.1016/j.wneu.2016.03.031 27001238

[7] Strand PS, Berntsen EM, Fyllingen EH et al (2021) Brain infarctions after glioma surgery: prevalence, radiological characteristics and risk factors. Acta Neurochir 163:3097–3108. 10.1007/s00701-021-04914-z PMC8520515 34468884

[8] Wada H, Shimauchi-Ohtaki H, Tosaka M et al (2024) Comparison of early postoperative diffusion-weighted magnetic resonance imaging findings after resection of gliomas and meningiomas. World Neurosurg 186:e296–e304. 10.1016/j.wneu.2024.03.126 38548056

[9] Berger A, Tzarfati G, Serafimova M et al (2022) Clinical and prognostic implications of rim restriction following glioma surgery. Sci Rep 12:1–10. 10.1038/s41598-022-16717-y PMC9329326 35896589

[10] Tsuchiya T, Takahashi M, Ohno M et al (2024) Delayed deep white matter ischemia after resection of gliomas by awake surgery. Neurosurg Pract 5:e00105. 10.1097/NP9.0000000000000105 PMC11809972 39959549

[11] Colen RR (2011) Magnetic resonance imaging appearance and changes on intracavitary Gliadel wafer placement: a pilot study. World J Radiol 3:266. 10.4329/wjr.v3.i11.266 PMC3226960 22132297

[12] Dörner L, Ulmer S, Rohr A, Mehdorn HM, Nabavi A (2011) Space-occupying cyst development in the resection cavity of malignant gliomas following Gliadel® implantation—incidence, therapeutic strategies, and outcome. J Clin Neurosci 18:347–351. 10.1016/j.jocn.2010.05.036 21237660

[13] Haim O, Agur A, Efrat OT, Valdes P, Ram Z, Grossman R (2023) The clinical significance of radiological changes associated with gliadel implantation in patients with recurrent high grade glioma. Sci Rep 13:11. 10.1038/s41598-022-27128-4 PMC9807577 36593342

[14] Bhattacharya K, Rai P, Moiyadi A et al (2025) Radiological hypertrophic olivary degeneration following posterior fossa tumor surgery. World Neurosurg 193:567–576. 10.1016/j.wneu.2024.10.043 39437889

[15] Stupp R, Mason WP, Van Den Bent MJ et al (2005) Radiotherapy plus concomitant and adjuvant temozolomide for glioblastoma. N Engl J Med 352:987–996. 10.1056/NEJMoa043330 15758009

[16] Weller M, Van Den Bent M, Preusser M et al (2021) EANO guidelines on the diagnosis and treatment of diffuse gliomas of adulthood. Nat Rev Clin Oncol 18:170–186. 10.1038/s41571-020-00447-z PMC7904519 33293629

[17] Zhang J, Stevens MFG, Bradshaw TD (2012) Temozolomide: mechanisms of action, repair and resistance. Curr Mol Pharmacol 5:102–114. 10.2174/1874467211205010102 22122467

[18] Hegi ME, Diserens AC, Gorlia T et al (2005) MGMT gene silencing and benefit from temozolomide in glioblastoma. N Engl J Med 352:997–1003. 10.1056/NEJMoa043331 15758010

[19] Brandes AA, Franceschi E, Tosoni A et al (2008) MGMT promoter methylation status can predict the incidence and outcome of pseudoprogression after concomitant radiochemotherapy in newly diagnosed glioblastoma patients. J Clin Oncol 26:2192–2197. 10.1200/JCO.2007.14.8163 18445844

[20] Wen PY, Macdonald DR, Reardon DA et al (2010) Updated response assessment criteria for high-grade gliomas: response assessment in Neuro-Oncology Working Group. J Clin Oncol 28:1963–1972. 10.1200/JCO.2009.26.3541 20231676

[21] Strauss SB, Meng A, Ebani EJ, Chiang GC (2019) Imaging glioblastoma posttreatment. Radiol Clin North Am 57:1199–1216. 10.1016/j.rcl.2019.07.003 31582045

[22] Brandsma D, Van Den Bent MJ (2009) Pseudoprogression and pseudoresponse in the treatment of gliomas. Curr Opin Neurol 22:633–638. 10.1097/WCO.0b013e328332363e 19770760

[23] Vollmuth P, Karschnia P, Sahm F, Park YW, Ahn SS, Jain R (2025) A radiologist’s guide to IDH-wildtype glioblastoma for efficient communication with clinicians: Part II—Essential information on post-treatment imaging. Korean J Radiol. 10.3348/kjr.2024.0983 PMC11955384 40015559

[24] Mullins ME, Barest GD, Schaefer PW, Hochberg FH, Gonzalez RG, Lev MH (2005) Radiation necrosis versus glioma recurrence: conventional MR imaging clues to diagnosis. AJNR Am J Neuroradiol 26:1967–1972 PMC8148818 16155144

[25] Wetzel EA, Farrell MJ, Eldred BSC et al (2023) Retrospective examination of pseudoprogression in IDH mutant gliomas. Neurooncol Adv 5:vdad028. 10.1093/noajnl/vdad028 PMC10148681 37128507

[26] Seyve A, Dehais C, Chinot O et al (2022) Incidence and characteristics of pseudoprogression in IDH-mutant high-grade gliomas: a POLA network study. Neuro Oncol. 10.1093/neuonc/noac194 PMC10013645 35953421

[27] Jaspers JPM, Taal W, van Norden Y et al (2023) Early and late contrast enhancing lesions after photon radiotherapy for IDH mutated grade 2 diffuse glioma. Radiother Oncol. 10.1016/j.radonc.2023.109674 37084885

[28] Esparragosa Vazquez I, Ndiaye M, Di Stefano AL et al (2023) T2-fluid-attenuated inversion recovery (FLAIR) pseudoprogression in patients with anaplastic oligodendrogliomas treated with procarbazine, lomustine and vincristine (PCV) chemotherapy alone. Eur J Neurol 30:2879–2883. 10.1111/ene.15873 37204066

[29] Garg K, Agrawal D (2023) Role of stereotactic radiosurgery in glial tumors. Neurol India 71:S207–S214. 10.4103/0028-3886.373633 37026354

[30] Cho NS, Hagiwara A, Eldred BSC et al (2022) Early volumetric, perfusion, and diffusion MRI changes after mutant isocitrate dehydrogenase (IDH) inhibitor treatment in IDH1-mutant gliomas. Neurooncol Adv 4:vdac124. 10.1093/noajnl/vdac124 PMC9400453 36033919

[31] Nowosielski M, Wiestler B, Goebel G et al (2014) Progression types after antiangiogenic therapy are related to outcome in recurrent glioblastoma. Neurology 82:1684–1692. 10.1212/WNL.0000000000000402 24727314

[32] Gatto L, Franceschi E, Tosoni A et al (2021) Distinct MRI pattern of ‘pseudoresponse’ in recurrent glioblastoma multiforme treated with regorafenib: case report and literature review. Clin Case Rep. 10.1002/ccr3.4604 PMC8380081 34457284

[33] Gaudino S, Marziali G, Giordano C et al (2022) Regorafenib in glioblastoma recurrence: how to deal with MR imaging treatments changes. Front Radiol 1:790456. 10.3389/fradi.2021.790456 PMC10365006 37492166

[34] Agarwal A, Desai A, Gupta V, Vibhute P (2022) Bevacizumab-induced coagulative necrosis with restricted diffusion. Radiol Imaging Cancer 4:e220089. 10.1148/rycan.220089 PMC9530776 36112037

[35] Nguyen HS, Milbach N, Hurrell SL et al (2016) Progressing bevacizumab-induced diffusion restriction is associated with coagulative necrosis surrounded by viable tumor and decreased overall survival in patients with recurrent glioblastoma. AJNR Am J Neuroradiol 37:2201–2208. 10.3174/ajnr.A4898 PMC5161572 27492073

[36] Werner JM, Wollring MM, Tscherpel C et al (2023) Multimodal imaging findings in patients with glioblastoma with extensive coagulative necrosis related to regorafenib. Neuro Oncol 25:1193–1195. 10.1093/neuonc/noad051 PMC10237410 36960770

[37] Liu X, Chen H, Tan G et al (2024) A comprehensive neuroimaging review of the primary and metastatic brain tumors treated with immunotherapy: current status, and the application of advanced imaging approaches and artificial intelligence. Front Immunol 15:1496627. 10.3389/fimmu.2024.1496627 PMC11634813 39669560

[38] Rhee JY, Ospina Botero JP, Nelson T et al (2026) Limited evidence of pseudoprogression following immune checkpoint inhibitor (ICI) therapy in glioblastoma. Neurooncol Adv 8:vdaf232. 10.1093/noajnl/vdaf232 PMC12850524 41613042

[39] Ageenko A, Vasileva N, Richter V, Kuligina E (2024) Combination of oncolytic virotherapy with different antitumor approaches against glioblastoma. Int J Mol Sci 25:2042. 10.3390/ijms25042042 PMC10889383 38396720

[40] Chawla S, Shehu V, Gupta PK, Nath K, Poptani H (2021) Physiological imaging methods for evaluating response to immunotherapies in glioblastomas. Int J Mol Sci 22:3867. 10.3390/ijms22083867 PMC8069140 33918043

[41] Chen H, Tan G, Zhong L et al (2025) MR perfusion characteristics of pseudoprogression in brain tumors treated with immunotherapy—a comparative study with chemo-radiation induced pseudoprogression and radiation necrosis. J Neurooncol 172:239–247. 10.1007/s11060-024-04910-0 PMC11832695 39688766

[42] Cuccarini V, Aquino D, Gioppo A et al (2019) Advanced MRI assessment during dendritic cell immunotherapy added to standard treatment against glioblastoma. J Clin Med 8:2007. 10.3390/jcm8112007 PMC6912338 31744235

[43] Hassankhani A, Bagley SJ, Dahmoush H, Parsons MS, Nabavizadeh A (2025) Role of neuroimaging in cancer-treatment neurotoxicity. AJR Am J Roentgenol 225:e2533139. 10.2214/AJR.25.33139 40557987

[44] Mahdi J, Dietrich J, Straathof K et al (2023) Tumor inflammation-associated neurotoxicity. Nat Med 29:803–810. 10.1038/s41591-023-02276-w PMC10166099 37024595

[45] Atsina KB, Sharan AD, Wu C et al (2017) JOURNAL CLUB: Longitudinal qualitative characterization of MRI features after laser interstitial thermal therapy in drug-resistant epilepsy. AJR Am J Roentgenol 208:48–56. 10.2214/AJR.16.16144 27657929

[46] Salem U, Kumar VA, Madewell JE et al (2019) Neurosurgical applications of MRI guided laser interstitial thermal therapy (LITT). Cancer Imaging 19:65. 10.1186/s40644-019-0250-4 PMC6792239 31615562

[47] Rademacher AF, Fadel HA, Pawloski JA et al (2025) Laser interstitial thermal therapy for intra-axial brain tumors: everything the neuroradiologist should know. AJNR Am J Neuroradiol 46:666–674. 10.3174/ajnr.A8427 PMC11979850 39572197

[48] Stupp R, Taillibert S, Kanner A et al (2017) Effect of tumor-treating fields plus maintenance temozolomide vs maintenance temozolomide alone on survival in patients with glioblastoma: a randomized clinical trial. JAMA 318:2306–2316. 10.1001/jama.2017.18718 PMC5820703 29260225

[49] Soni VS, Yanagihara TK (2019) Tumor treating fields in the management of glioblastoma: opportunities for advanced imaging. Cancer Imaging 19:76. 10.1186/s40644-019-0259-8 PMC6884888 31783910

[50] Iwabuchi S (2024) Delayed pseudoprogression in glioblastoma patients treated with tumor-treating fields. Cureus 16:e55147. 10.7759/cureus.55147 PMC10979818 38558596

[51] Iv M, Naya L, Sanan S et al (2024) Tumor treating fields increases blood–brain barrier permeability and relative cerebral blood volume in patients with glioblastoma. Neuroradiol J 37:107–118. 10.1177/19714009231207083 PMC10863570 37931176

[52] Chang KW, Hong SW, Chang WS, Jung HH, Chang JW (2023) Characteristics of focused ultrasound mediated blood-brain barrier opening in magnetic resonance images. J Korean Neurosurg Soc 66:172–182. 10.3340/jkns.2022.0236 PMC10009247 36537034

[53] Nabavizadeh A, Narsinh K, Kaufmann TJ et al (2026) Focused ultrasound in brain tumors: mechanisms, imaging guidance, and emerging clinical applications. AJNR Am J Neuroradiol. 10.3174/ajnr.A9126 PMC13232671 41360502

[54] Romano A, Moltoni G, Blandino A et al (2023) Radiosurgery for brain metastases: challenges in imaging interpretation after treatment. Cancers (Basel) 15:5092. 10.3390/cancers15205092 PMC10605307 37894459

[55] Lasocki A, Sia J, Stuckey SL (2025) Differentiation between radiation necrosis and true tumour progression after radiotherapy to intracranial metastases. J Med Imaging Radiat Oncol 69:414–424. 10.1111/1754-9485.13847 PMC12120592 40057844

[56] Salbas A, Koc AM, Coskun M, Horoz EM, Sengul A, Gelal MF (2025) Temporal evolution of MRI findings and survival outcomes in patients with brain metastases after stereotactic radiosurgery. BMC Med Imaging 25:161. 10.1186/s12880-025-01713-1 PMC12080185 40369456

[57] Patel TR, McHugh BJ, Bi WL, Minja FJ, Knisely JPS, Chiang VL (2011) A comprehensive review of MR imaging changes following radiosurgery to 500 brain metastases. AJNR Am J Neuroradiol 32:1885–1892. 10.3174/ajnr.A2668 PMC7966021 21920854

[58] Digernes I, Grøvik E, Nilsen LB et al (2018) Brain metastases with poor vascular function are susceptible to pseudoprogression after stereotactic radiation surgery. Adv Radiat Oncol 3:559–567. 10.1016/j.adro.2018.05.005 PMC6200880 30370356

[59] Alattar AA, Carroll K, Hirshman BR, Joshi RS, Sanghvi P, Chen CC (2018) Cystic formation after stereotactic radiosurgery of brain metastasis. World Neurosurg 114:e719–e728. 10.1016/j.wneu.2018.03.066 29551723

[60] Kubo K, Kenjo M, Doi Y et al (2019) MRI appearance change during stereotactic radiotherapy for large brain metastases and importance of treatment plan modification during treatment period. Jpn J Radiol 37:850–859. 10.1007/s11604-019-00886-4 PMC6874519 31617151

[61] Maksoud Z, Schmidt MA, Huang Y et al (2022) Transient enlargement in meningiomas treated with stereotactic radiotherapy. Cancers (Basel) 14:1547. 10.3390/cancers14061547 PMC8946188 35326697

[62] Rueß D, Schütz B, Celik E et al (2023) Pseudoprogression of vestibular schwannoma after stereotactic radiosurgery with Cyberknife®: proposal for new response criteria. Cancers (Basel) 15:1496. 10.3390/cancers15051496 PMC10000564 36900290

[63] Katsura M, Sato J, Akahane M, Furuta T, Mori H, Abe O (2021) Recognizing radiation-induced changes in the central nervous system: where to look and what to look for. Radiographics 41:224–248. 10.1148/rg.2021200064 33216673

[64] Babin M, Golse M, Khaterchi M et al (2025) Perivascular enhancement pattern: identification, diagnostic spectrum and practical approach—a pictorial review. J Neuroradiol 52:101242. 10.1016/j.neurad.2025.101242 39828213

[65] Asano K, Fujiwara N, Katayama K et al (2026) New MRI findings of late delayed radiation injuries in long-term survivors after radiotherapy: punctate enhancing dot-, oval-, or rod-like lesions. Neuroradiology. 10.1007/s00234-026-03994-x 41979659

[66] Nishioka K, Kawamura M, Iima M et al (2025) The glymphatic system in oncology: from the perspective of a radiation oncologist. J Radiat Res. 10.1093/jrr/rraf027 PMC12283521 40460451

[67] Black DF, Morris JM, Lindell EP et al (2013) Stroke-like migraine attacks after radiation therapy (SMART) syndrome is not always completely reversible: a case series. AJNR Am J Neuroradiol 34:2298–2303. 10.3174/ajnr.A3602 PMC7965219 23788601

[68] Singh TD, Hajeb M, Rabinstein AA et al (2021) SMART syndrome: retrospective review of a rare delayed complication of radiation. Eur J Neurol 28:1316–1323. 10.1111/ene.14632 33159349

[69] Ota Y, Leung D, Lin E et al (2022) Prognostic factors of stroke-like migraine attacks after radiation therapy (SMART) syndrome. AJNR Am J Neuroradiol 43:396–401. 10.3174/ajnr.A7424 PMC8910816 35177545

[70] Di Stefano AL, Berzero G, Ducray F et al (2019) Stroke-like events after brain radiotherapy: a large series with long-term follow-up. Eur J Neurol 26:639–650. 10.1111/ene.13870 30471162

[71] Rheims S, Ricard D, Van Den Bent M et al (2011) Peri-ictal pseudoprogression in patients with brain tumor. Neuro Oncol 13:775–782. 10.1093/neuonc/nor082 PMC3129278 21727213

[72] Amseian G, Aya F, Pineda C et al (2025) Assessing brain metastasis response to immunotherapy: a pictorial review of atypical responses and intracranial adverse events. Insights Imaging 16:258. 10.1186/s13244-025-02125-z PMC12630472 41258375

[73] Galldiks N, Kocher M, Ceccon G et al (2020) Imaging challenges of immunotherapy and targeted therapy in patients with brain metastases: response, progression, and pseudoprogression. Neuro Oncol 22:17–30. 10.1093/neuonc/noz147 PMC6954406 31437274

[74] Govaerts CW, Kramer MCA, Bosma I et al (2025) Incidence and clinical features of pseudoprogression in brain metastases after immune-checkpoint inhibitor therapy: a retrospective study. Cancers (Basel) 17:2425. 10.3390/cancers17152425 PMC12346240 40805128

[75] Urban H, Steidl E, Hattingen E et al (2022) Immune checkpoint inhibitor-induced cerebral pseudoprogression: patterns and categorization. Front Immunol 12:798811. 10.3389/fimmu.2021.798811 PMC8761630 35046955

[76] Kersch CN, Ambady P, Hamilton BE, Barajas RF (2022) MRI and PET of brain tumor neuroinflammation in the era of immunotherapy, from the *AJR* Special Series on Inflammation. AJR Am J Roentgenol 218:582–596. 10.2214/AJR.21.26159 PMC9134929 34259035

[77] Lasocki A, Spain L (2025) Neuroimaging features of immune-related adverse events due to immune checkpoint inhibitor therapy. Insights Imaging 16:120. 10.1186/s13244-025-01999-3 PMC12162444 40506672

[78] Kurokawa R, Ota Y, Gonoi W et al (2020) MRI findings of immune checkpoint inhibitor–induced hypophysitis: possible association with fibrosis. AJNR Am J Neuroradiol 41:1683–1689. 10.3174/ajnr.A6692 PMC7583108 32763900

[79] Ibáñez-Juliá MJ, Bataller L, Cabello-Murgui FJ et al (2025) Clinical and radiological features of pseudoprogression in brain tumors treated with immune checkpoint inhibitors. J Neurooncol 174:779–788. 10.1007/s11060-025-05091-0 PMC12263762 40426008

[80] Srinivasan ES, Grabowski MM, Nahed BV, Barnett GH, Fecci PE (2021) Laser interstitial thermal therapy for brain metastases. Neurooncol Adv 3:v16–v25. 10.1093/noajnl/vdab128 PMC8633752 34859229

[81] Chu HH, Choi SH, Ryoo I et al (2013) Differentiation of true progression from pseudoprogression in glioblastoma treated with radiation therapy and concomitant temozolomide: comparison study of standard and high-b-value diffusion-weighted imaging. Radiology 269:831–840. 10.1148/radiol.13122024 23771912

[82] Hu R, Hoch MJ (2021) Application of diffusion weighted imaging and diffusion tensor imaging in the pretreatment and post-treatment of brain tumor. Radiol Clin North Am. 10.1016/j.rcl.2021.01.003 33926681

[83] Liao D, Liu YC, Liu JY, Wang D, Liu XF (2023) Differentiating tumour progression from pseudoprogression in glioblastoma patients: a monoexponential, biexponential, and stretched-exponential model-based DWI study. BMC Med Imaging 23:119. 10.1186/s12880-023-01082-7 PMC10494379 37697237

[84] Kopřivová T, Keřkovský M, Jůza T et al (2024) Possibilities of using multi-b-value diffusion magnetic resonance imaging for classification of brain lesions. Acad Radiol 31:261–272. 10.1016/j.acra.2023.10.002 37932166

[85] Li AY, Iv M (2022) Conventional and advanced imaging techniques in post-treatment glioma imaging. Front Radiol 2:883293. 10.3389/fradi.2022.883293 PMC10365131 37492665

[86] Van Dijken BRJ, Van Laar PJ, Smits M, Dankbaar JW, Enting RH, Van Der Hoorn A (2019) Perfusion MRI in treatment evaluation of glioblastomas: clinical relevance of current and future techniques. J Magn Reson Imaging 49:11–22. 10.1002/jmri.26306 PMC6590309 30561164

[87] Herings SDA, Van Der Wijk MW, Von Beckerath V et al (2024) Fractional tumor burden maps increase the confidence of reading brain MR perfusion. Eur J Radiol 178:111644. 10.1016/j.ejrad.2024.111644 39084028

[88] Hilario A, Salvador E, Cardenas A et al (2024) Low rCBV values in glioblastoma tumor progression under chemoradiotherapy. Neuroradiology 66:317–323. 10.1007/s00234-023-03279-7 38183424

[89] Kim M, Park JE, Emblem K, Bjørnerud A, Kim HS (2021) Vessel type determined by vessel architectural imaging improves differentiation between early tumor progression and pseudoprogression in glioblastoma. AJNR Am J Neuroradiol 42:663–670. 10.3174/ajnr.A6984 PMC8041004 33541891

[90] Jing H, Yan X, Li J, Qin D, Zhang N, Zhang H (2022) The value of dynamic contrast-enhanced magnetic resonance imaging (DCE-MRI) in the differentiation of pseudoprogression and recurrence of intracranial gliomas. Contrast Media Mol Imaging 2022:5680522. 10.1155/2022/5680522 PMC9337951 35935318

[91] Kim KJ, Park M, Joo B, Ahn SJ, Suh SH (2022) Dynamic contrast-enhanced MRI and its applications in various central nervous system diseases. Invest Magn Reson Imaging 26:256. 10.13104/imri.2022.26.4.256

[92] Jaafar N, Alsop DC (2024) Arterial spin labeling: key concepts and progress towards use as a clinical tool. Magn Reson Med Sci 23:352–366. 10.2463/mrms.rev.2024-0013 PMC11234948 38880616

[93] Alsulami TA, Hyare H, Thomas DL, Golay X (2023) The value of arterial spin labelling (ASL) perfusion MRI in the assessment of post-treatment progression in adult glioma: a systematic review and meta-analysis. Neurooncol Adv 5:vdad122. 10.1093/noajnl/vdad122 PMC10576519 37841694

[94] Sidibe I, Tensaouti F, Roques M, Cohen-Jonathan-Moyal E, Laprie A (2022) Pseudoprogression in glioblastoma: role of metabolic and functional MRI—systematic review. Biomedicines. 10.3390/biomedicines10020285 PMC8869397 35203493

[95] El-Abtah ME, Talati P, Fu M et al (2022) Magnetic resonance spectroscopy outperforms perfusion in distinguishing between pseudoprogression and disease progression in patients with glioblastoma. Neurooncol Adv 4:vdac128. 10.1093/noajnl/vdac128 PMC9446677 36071927

[96] Zhou J (2011) Amide proton transfer imaging of the human brain. Methods Mol Biol 711:227–237. 10.1007/978-1-61737-992-5_10 PMC3547598 21279604

[97] Ma B, Blakeley JO, Hong X et al (2016) Applying amide proton transfer-weighted MRI to distinguish pseudoprogression from true progression in malignant gliomas. J Magn Reson Imaging 44:456–462. 10.1002/jmri.25159 PMC4946988 26788865

[98] Essed RA, Prysiazhniuk Y, Wamelink IJ, Azizova A, Keil VC (2024) Performance of amide proton transfer imaging to differentiate true progression from therapy-related changes in gliomas and metastases. Eur Radiol 35:580–591. 10.1007/s00330-024-11004-y PMC11782315 39134744

[99] Li Y, Ma Y, Wu Z et al (2021) Advanced imaging techniques for differentiating pseudoprogression and tumor recurrence after immunotherapy for glioblastoma. Front Immunol. 10.3389/fimmu.2021.790674 PMC8656432 34899760

[100] Hoggarth AR, Muthukumar S, Thomas SM, Crowley J, Kiser J, Witcher MR (2024) Clinical theranostics in recurrent gliomas: a review. Cancers (Basel) 16:1715. 10.3390/cancers16091715 PMC11083317 38730666

[101] Villanueva-Meyer JE, Bakas S, Tiwari P et al (2024) Artificial intelligence for response assessment in neuro oncology (AI-RANO), part 1: review of current advancements. Lancet Oncol 25:e581–e588. 10.1016/S1470-2045(24)00316-4 PMC12045294 39481414

[102] Garcia-Rizk JA, Ortiz Haro MF, Santos Aragon LN, De La Mata-Moya D, Hernandez Bojorquez M (2024) Magnetic resonance imaging assessment of morphological changes and molecular behavior to evaluate treatment response of brain metastatic lesions after stereotactic radiosurgery. Cureus 16:e73630. 10.7759/cureus.73630 PMC11645163 39677170

[103] Hainc N, Alsafwani N, Gao A et al (2021) The centrally restricted diffusion sign on MRI for assessment of radiation necrosis in metastases treated with stereotactic radiosurgery. J Neurooncol 155:325–333. 10.1007/s11060-021-03879-4 PMC8651583 34689307

[104] Cha J, Kim ST, Kim HJ et al (2013) Analysis of the layering pattern of the apparent diffusion coefficient (ADC) for differentiation of radiation necrosis from tumour progression. Eur Radiol 23:879–886. 10.1007/s00330-012-2638-4 22903642

[105] Chen Z, Zu J, Li L, Lu X, Ni J, Xu J (2017) Assessment of stereotactic radiosurgery treatment response for brain metastases using MRI based diffusion index. Eur J Radiol Open 4:84–88. 10.1016/j.ejro.2017.06.002 PMC5506876 28725661

[106] Chernov MF, Hayashi M, Izawa M et al (2009) Dynamics of metabolic changes in intracranial metastases and distant normal-appearing brain tissue after stereotactic radiosurgery: a serial proton magnetic resonance spectroscopy study. Neuroradiol J 22:58–71. 10.1177/197140090902200109 24206954

[107] Admojo L, Korte J, Anderson N, Phillips C, Caspersz L, Lasocki A (2023) Investigating the role of delayed contrast magnetic resonance imaging (MRI) to differentiate radiation necrosis from tumour recurrence in brain metastases after stereotactic radiosurgery. J Med Imaging Radiat Oncol 67:292–298. 10.1111/1754-9485.13504 36650724

[108] Heyn C, Bishop J, Moody AR et al (2025) Gadolinium-enhanced T2 FLAIR is an imaging biomarker of radiation necrosis and tumor progression in patients with brain metastases. AJNR Am J Neuroradiol 46:129–135. 10.3174/ajnr.A8431 PMC11735435 39107039

[109] Gonzalez AT, Haskell-Mendoza AP, Reason EH et al (2025) Identifying predictive factors for radiation necrosis vs local recurrence in biopsy-proven enlarging lesions post-stereotactic radiosurgery for brain metastases. Neurooncol Pract. 10.1093/nop/npaf125 PMC13268959 42312117

