# Supplementary material for: Post-therapy imaging of adult brain tumours: patterns, pitfalls, and practical framework
Source: Insights Imaging. 2026 Sep 19;17:231. doi: 10.1186/s13244-026-02398-y (PMC13589733; doi:10.1186/s13244-026-02398-y)
Supplement: Supplementary file 1 [file 13244_2026_2398_MOESM1_ESM.pdf]

Post-Therapy Imaging of Adult Brain Tumours: Patterns, Pitfalls, and Practical Framework

ELECTRONIC SUPPLEMENTARY MATERIAL

Supplementary information

Additional file 1. Interactive treatment- and time-based framework for post-therapy brain tumour imaging (HTML).

An interactive version of Fig. 12. The reader selects a treatment category and time point to display the corresponding expected imaging patterns, with an accompanying description of each. Abbreviations are defined within the file. Provided as an educational adjunct; no patient data are included.
