# Supplementary material for: Post-therapy imaging of adult brain tumours: patterns, pitfalls, and practical framework
Source: Insights Imaging. 2026 Sep 19;17:231. doi: 10.1186/s13244-026-02398-y (PMC13589733; doi:10.1186/s13244-026-02398-y)
Supplement: Supplementary file 2 [file 13244_2026_2398_MOESM2_ESM.html]

Post-Therapy Adult Brain Tumour Imaging — Treatment × Time Matrix

# Post-Therapy Adult Brain Tumour Imaging

Treatment × time matrix — choose a tumour type, a treatment, and a timepoint.

Electronic supplementary material

Adult diffuse glioma
Brain metastases

Time since treatment reference point

0

**Note.** Timing is referenced to each treatment's own starting point — resection for surgery, completion of radiotherapy for radiotherapy-based regimens, and start of treatment for systemic and device therapies. Intervals are approximate and representative. Use the clinical scenario, treatment field, baseline study, and interval evolution before assigning likelihood; this matrix is a guide, not a diagnostic threshold.

**Abbreviations.** ADC apparent diffusion coefficient · BBB blood–brain barrier · CAR-T chimeric antigen receptor T-cell · CCRT concurrent chemoradiotherapy · DCE dynamic contrast-enhanced · DSC dynamic susceptibility contrast · FLAIR fluid-attenuated inversion recovery · GBM glioblastoma · ICI immune checkpoint inhibitor · IDH isocitrate dehydrogenase · iPsP immunotherapy-induced pseudoprogression · K^trans^ volume transfer constant · LITT laser interstitial thermal therapy · MGMT O⁶-methylguanine-DNA methyltransferase · MRI magnetic resonance imaging · PCV procarbazine, lomustine, and vincristine · PsP pseudoprogression · rCBV (nrCBV) (normalised) relative cerebral blood volume · RT radiotherapy · SMART stroke-like migraine attacks after radiation therapy · SRS stereotactic radiosurgery · SWI susceptibility-weighted imaging · TIAN tumour inflammation-associated neurotoxicity · TMZ temozolomide · TTFields tumour-treating fields.
